# Freeform Fabrication of Layered Halide Perovskite Nanowire Heterojunctions

**DOI:** 10.1002/adma.202522768

**Published:** 2026-03-04

**Authors:** Sixi Cao, Yu Liu, Zhuofei Gan, Shiqi Hu, Jihyuk Yang, Zhuoran Wang, Mingjian Yuan, Wen‐Di Li, Ji Tae Kim

**Affiliations:** ^1^ Department of Mechanical Engineering The University of Hong Kong Hong Kong China; ^2^ State Key Laboratory of Advanced Chemical Power Sources Key Laboratory of Advanced Energy Materials Chemistry (Ministry of Education) Frontiers Science Center for New Organic Matter College of Chemistry Nankai University Tianjin China; ^3^ Department of Mechanical Engineering Korea Advanced Institute of Science and Technology (KAIST) Daejeon Republic of Korea

**Keywords:** additive manufacturing, layered perovskites, nanowire heterojunction, optoelectronics

## Abstract

Layered halide perovskites assembled into heterostructures offer precise manipulation of interfaces and electronic structures, making them ideal candidates for a wide range of optoelectronic applications such as photovoltaics, light‐emitting diodes, lasers, and photodetectors. Here, we demonstrate freeform fabrication of layered halide perovskite nanowire heterojunctions with programmed geometry and composition. The method utilizes a femtoliter meniscus of precursor solution to draw a path of solution‐mediated layered halide perovskite crystallization at will, enabling the deterministic assembly of freestanding, multi‐segmented nanowire heterojunctions. The interdiffusion kinetics of halide anions across the layered halide perovskite nanowire heterojunctions have been investigated, revealing suppressed ion migration and enhanced stability compared to their 3D perovskite counterparts. Furthermore, we demonstrate proof‐of‐concept self‐powered photodetectors that exhibit rectification, zero‐bias photocurrent, and fast response, directly validating the functional translation of structural programmability. This work establishes femtoliter meniscus‐guided nanoprinting as a versatile and scalable platform for programmable optoelectronic nanodevices.

## Graphical abstract

‘All‐at‐once’ freeform fabrication of layered perovskite nanowire heterojunctions is realized by 3D printing employing meniscus‐guided crystallization. The integration of nanoscale additive manufacturing with layered halide perovskites will provide a versatile platform to freely design and realize highly stable perovskite‐based optoelectronic integrated circuits.

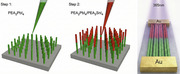

## 1 Introduction

The integration of materials into heterostructures, enabling precise control of interfaces and electronic structures, is critical for the advancement of modern electronics and optoelectronics [1, 2, 3]. Among emerging candidates, layered halide perovskites have attracted intense interest owing to their unique quantum‐well architecture, which combines outstanding optoelectronic properties with intrinsic structural stability and solution processability [4, 5, 6]. When assembled into heterostructures, these quantum‐well–like materials exhibit remarkable tolerance to lattice mismatch and inherently suppressed halide ion diffusion at interfaces, distinguishing them from conventional 3D perovskites [7, 8, 9]. Such features expand their potential in next‐generation devices, including photovoltaics [10], self‐powered photodetectors [11], multicolor displays [12], and encrypted light communications [13].

To date, layered halide perovskite heterostructures have been fabricated via vapor deposition [14, 15], solution‐phase synthesis [11, 12, 16], dry transfer [17, 18], anion exchange [19], and mechanical exfoliation combined with kinetic Wulff‐shaped growth [20]. However, these methods predominantly yield planar thin films, with limited ability to program complex 3D morphologies. Controlling heterostructure morphology, particularly in three dimensions, offers remarkable advantages [21]. Among various nanostructures, nanowires are particularly notable for their efficient charge transport and reduced recombination losses [22]. Therefore, constructing layered halide perovskite heterostructures within nanowire geometries is expected to enhance charge separation and collection efficiency in optoelectronic devices [23, 24, 25].

Recent progress has enabled the fabrication of layered perovskite nanowires and their heterostructures through solution method [26], vapor‐phase growth [27, 28], or lithographic templating [29, 30]. However, these methods continue to suffer from limited control over nanowire dimensions, position, and composition, restricting their seamless integration into functional circuits [31].

Here, we report meniscus‐guided 3D nanoprinting of layered halide perovskite nanowire heterostructures, enabling deterministic assembly of freestanding, multi‐segmented nanowires with programmable geometry and sharp, well‐defined interfaces. Unlike prior demonstrations in thin films or single‐phase nanowires, our approach integrates multiple compositions into a single programmable nanowire, yielding freeform nanowire heterojunctions that are difficult to achieve via conventional growth methods. By combining spatially resolved photoluminescence mapping with Boltzmann–Matano (BM) analysis, we quantitatively resolve halide ion migration across heterointerfaces—demonstrating markedly suppressed diffusion in layered perovskite nanowire heterostructures compared to their 3D counterparts. A key distinction from thin‐film studies [32, 33] is that the vertically aligned nanowires provide an intrinsically 1D transport pathway, enabling BM analysis in a rigorous and reproducible manner. Finally, we demonstrate proof‐of‐concept self‐powered photodetectors based on the printed nanowire heterostructures, directly linking nanoscale programmability and interfacial stability to device‐level optoelectronic performance. These advances position meniscus‐guided nanoprinting as a powerful strategy for realizing stable, programmable nanostructures that can be seamlessly integrated into next‐generation optoelectronic nanodevices.

## 2 Results and Discussion

### 2.1 3D Printing of Nanowire Heterostructures

**FIGURE 1:**
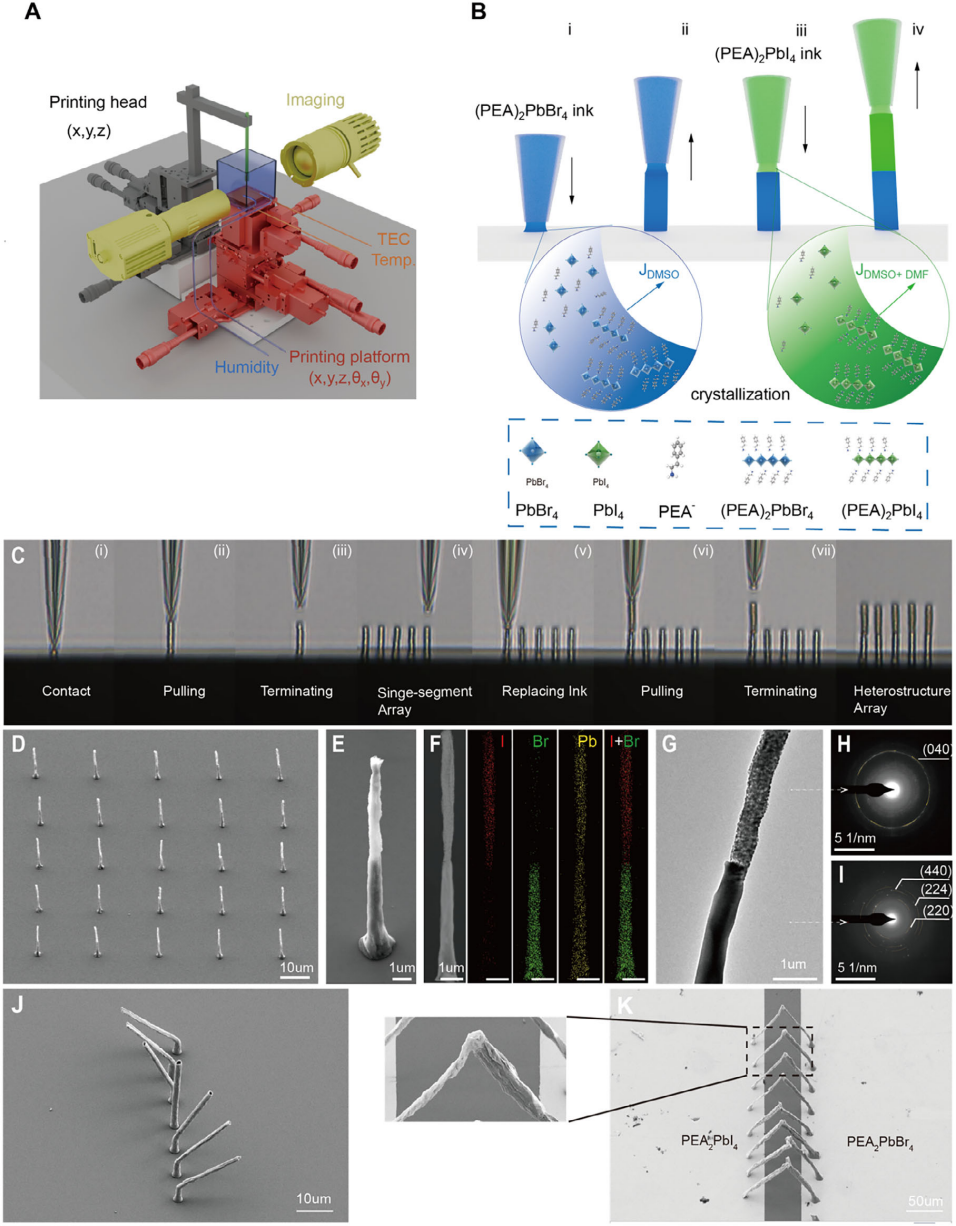
Femtoliter meniscus‐guided 3D printing of layered halide perovskite nanowire heterostructures. (A) Schematic illustration of the custom‐built 3D nanoprinting platform integrating nanopipette positioning with environmental control. (B) Stepwise printing process of a freestanding (PEA)_2_PbBr_4_/(PEA)_2_PbI_4_ nanowire heterostructure: (i) formation of a femtoliter meniscus of precursor ink between the pipette and substrate; (ii) directional crystallization of a (PEA)_2_PbBr_4_ nanowire via solvent‐evaporation–driven meniscus guidance; (iii) replacement with iodide precursor ink; and (iv) sequential printing of a (PEA)_2_PbI_4_ segment, yielding a sharp heterointerface. (C) Optical micrographs directly capturing the sequential growth of a nanowire heterostructure array. (D,E) FE‐SEM images of a 5 × 5 freestanding heterostructure array (D) and an individual nanowire (E), confirming large‐area reproducibility and uniformity. (F) TEM image and corresponding EDS elemental mappings of I, Br, and Pb, showing sharp spatial segregation of halides across the heterointerface. (G–I) Bright‐field TEM image (G) and SAED patterns of (H) (PEA)_2_PbI_4_ and (I) (PEA)_2_PbBr_4_ segments, verifying phase purity and polycrystallinity. (J) SEM image of inclined nanowires produced by controlling the meniscus‐guiding direction, demonstrating programmable structural versatility. (K) FE‐SEM image of suspended nanowire heterostructures integrated directly between prepatterned metal electrodes.

Figure 1A shows the femtoliter meniscus‐guided 3D nanoprinting platform developed in this work. The system integrates sub‐micrometer motion control with an environmentally regulated chamber, where temperature and humidity are precisely controlled to ensure a reproducible printing environment. Figure 1B schematically illustrates the stepwise printing process. A femtoliter meniscus is formed at the pipette tip, and upon contacting the substrate, the meniscus bridges the pipette and surface (Figure 1B i). Meniscus stability is maintained by the balance of interfacial forces at the three‐phase contact line, following the Neumann quadrilateral relation. Retracting the pipette at a controlled speed of 2–20 µm s^−^^1^ (Figure 1B ii), promotes solvent evaporation and progressively concentrates the precursor within the meniscus. Once the local solute concentration exceeds the temperature‐dependent solubility limit, supersaturation arises and provides the chemical potential that drives crystallization. Continuous nanowire growth is governed by the balance between this evaporation‐induced crystallization rate and the pulling speed. Growth is terminated by abruptly accelerating the pipette to ≈200 µm s^−^^1^, which ruptures the meniscus. The vertical placement resolution was set to 0.10 µm/step in our setup. These coupled constraints define the operational window for stable, reproducible nanowire printing (Figures S1).

To program heterostructures, we sequentially exchange micropipettes preloaded with different precursor solutions. The nanowire can be programmably extended with distinct compositions and sharp interfaces (Figure 1B iii,iv)—exemplified here by (PEA)_2_PbBr_4_/(PEA)_2_PbI_4_ nanowires. Alignment accuracy during pipette exchange was addressed by returning the stage to predefined coordinates, followed by microscope‐assisted registration to previously printed features. Optical micrographs (Figure 1C) provide real‐time visualization of this process, capturing continuous and well‐aligned rows of nanowire heterostructures. These results highlight the stability and reproducibility of the printing process and validate its programmable nature. A representative video is provided in Movie S1.

Reproducibility was further established by printing a 5 × 5 array of nanowire heterostructures with a 20 µm pitch (Figure 1D; Movie S2). A high‐resolution field‐emission scanning electron microscopy (FE‐SEM) image of individual nanowires (Figure 1E) shows sharp segment junctions, while energy‐dispersive X‐ray spectroscopy (EDS) mapping (Figure 1F) confirms uniform Pb distribution with Br and I spatially segregated into opposite segments. Crystallographic information was obtained by transmission electron microscopy (TEM), enabled by directly printing heterojunction nanowires onto TEM grids without alignment or transfer. The bright‐field image in Figure 1G also reveals structural continuity across the interface. Selected‐area electron diffraction (SAED) patterns of (PEA)_2_PbI_4_ and (PEA)_2_PbBr_4_ (Figure 1H,I) display distinct diffraction rings indexed to the (040) reflection of the iodide and the (440), (224), and (220) reflections of the bromide, confirming the polycrystalline nature and phase identity of the printed nanowires.

Arrays of nanowires with controlled heights between 10 and 30 µm (Figure S4A) demonstrate reproducible vertical growth, while the aspect ratio of single nanowires is highly tunable and can readily exceed 200 for lengths over 100 µm (Figure S4C). Representative arrays of 50 nanowires (Figure S5) further confirm reliable vertical‐direction programmability within the precision of the stage. Moreover, our method affords structural versatility, enabling omnidirectional trajectories and programmable inclination angles for fully 3D architectures (Figure 1J). The tubular appearance in Figure 1J can be explained by intrinsic crystallization dynamics during meniscus‐guided growth [34]. This spatial freedom markedly expands the design space for functional nanoscale architectures. Direct integration of suspended heterostructures between prepatterned electrodes (Figure 1K) further demonstrates seamless device‐level incorporation. While the current implementation is serial, throughput would benefit from automated exchange workflows and parallelization strategies (e.g., multi‐pipette arrays), without altering the confined‐meniscus growth principle.

### 2.2 Optimization of Printing Parameters

**FIGURE 2:**
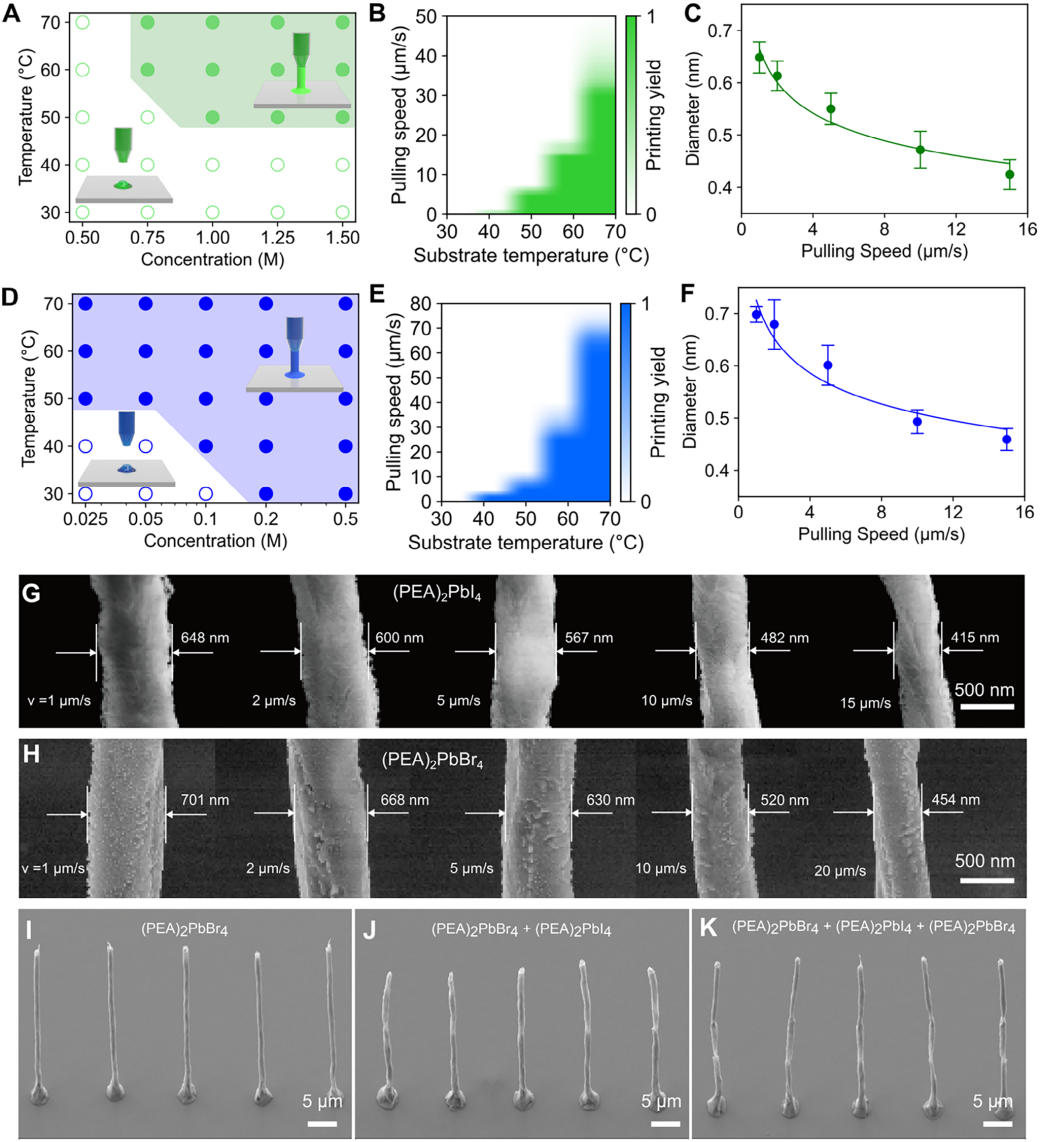
Optimization of femtoliter meniscus‐guided 3D printing for layered halide perovskite nanowires. (A,D) Printable concentration–temperature phase spaces for uniform nanowire growth of (A) (PEA)_2_PbI_4_ and (D) (PEA)_2_PbBr_4_ inks (DMF: DMSO = 4:1) at a pulling speed of 2 µm s^−^^1^, revealing distinct halide‐dependent solubility regimes. (B,E) Printing yield maps for (B) (PEA)_2_PbI_4_ (1.25 m) and (E) (PEA)_2_PbBr_4_ (0.10 m) nanowires as functions of pulling speed and substrate temperature, showing the operational windows for continuous nanowire growth. (C,F) Diameter–velocity relationships for (C) (PEA)_2_PbI_4_ and (F) (PEA)_2_PbBr_4_ nanowires at 60°C, exhibiting inverse dependence consistent with mass‐flow continuity. (G,H) FE‐SEM images confirming systematic diameter reduction of (G) (PEA)_2_PbI_4_ and (H) (PEA)_2_PbBr_4_ nanowires with increasing pulling speed, demonstrating deterministic dimensional control. (I–K) Representative FE‐SEM images of (I) single‐, (J) dual‐, and (K) triple‐segmented nanowire heterostructure arrays, highlighting the capability of sequential meniscus‐guided printing to integrate multiple compositions with high structural fidelity.

In meniscus‐guided printing, solvent evaporation induces convective flow and supersaturation, driving crystallization within the liquid bridge [35]. Continuous nanowire growth requires maintaining a stable meniscus at the pipette–substrate interface, where pulling speed must balance the intrinsic crystal growth rate [36]. Consequently, the accessible processing window is governed by the coupled effects of temperature, precursor concentration, and pulling speed. To establish the operational window for layered halide perovskites, we systematically mapped the concentration–temperature and speed–temperature phase diagrams for (PEA)_2_PbI_4_ and (PEA)_2_PbBr_4_ nanowires (Figure 2), while maintaining the relative humidity below 10% to suppress moisture‐induced defects [37].

As shown in Figure 2A,D, (PEA)_2_PbI_4_ achieves continuous growth within 1.0–1.5 m and 50°C–70°C, whereas (PEA)_2_PbBr_4_ crystallizes efficiently across 0.10–0.50 m under similar conditions. This distinction arises from halide‐dependent solubility and supersaturation in DMF/DMSO, with bromide systems more readily reaching supersaturation at lower bulk concentrations [38]. At suboptimal conditions (e.g., <1.0 m or <50°C for iodides; <0.10 m or <40°C for bromides), the nanowire yields decline sharply due to insufficient growth, whereas excessively high concentrations or temperatures accelerate supersaturation, leading to uncontrolled nucleation and morphological instabilities. In practice, the overall printing success rate was influenced by meniscus stability and the reproducibility of segment switching, with occasional failures such as tip clogging. Within these operational windows, 1.25 m for (PEA)_2_PbI_4_ and 0.10 m for (PEA)_2_PbBr_4_ were selected as representative optimal conditions, balancing crystal growth kinetics and interface quality to reproducibly yield uniform nanowires over a broad temperature range.

At these optimal precursor concentrations, the accessible printing window is further defined by the coupled effects of pulling speed and temperature. As solvent evaporation accelerates at elevated temperatures, the crystal growth rate increases, thereby raising the maximum pulling speed that permits continuous growth (defined here as the threshold speed). Figure 2B,E shows that the threshold increases systematically with temperature, consistent with accelerated crystallization dynamics. Exceeding the threshold causes the meniscus to rupture and halts printing, while excessively high temperatures increase clogging risk due to uncontrolled supersaturation. A printing temperature of 60°C was therefore selected as a practical compromise between process stability and operational reliability.

Beyond ensuring growth continuity, precise control over nanowire diameter is equally critical for achieving high‐quality nanowire heterostructures, as dictated by mass‐flow continuity during crystallization. Under the assumption of steady‐state growth, the mass flow rate is given by [39].

(1) $$m\left(v\right)=\rho_{s}\pi {\left(\frac{D}{2}\right)}^{2}v$$

where *m*(*v*) is the mass flow rate as a function of *v*, ρ_*s*_ is the nanowire density, *D* is the nanowire diameter, and *v* is the pulling speed. This relation predicts an inverse scaling of diameter with pulling speed. The experimentally measured *D–v* relations for (PEA)_2_PbI_4_ and (PEA)_2_PbBr_4_ nanowires (Figure 2C,F) follow this trend, with fitted power‐law dependences *D* = 0.66 *v* ^−0.15^ and *D* = 0.73 *v* ^−0.15^, respectively. The corresponding FE‐SEM images (Figure 2G,H) further corroborate the systematic decrease in diameter with increasing pulling speed.

With these optimized parameters, we fabricated single‐, double‐, and triple‐segment nanowire arrays with high structural uniformity (Figure 2I–K). The ability to reproducibly tailor nanowire dimensions while seamlessly integrating multiple material segments in a single‐step process underscores the robustness of femtoliter meniscus‐guided 3D printing. This capability is particularly powerful for constructing multi‐heterojunction architectures—a level of structural complexity that remains challenging to realize using conventional bottom‐up or top‐down approaches.

### 2.3 Library of Layered Halide Perovskite Nanowire Heterostructures

**FIGURE 3:**
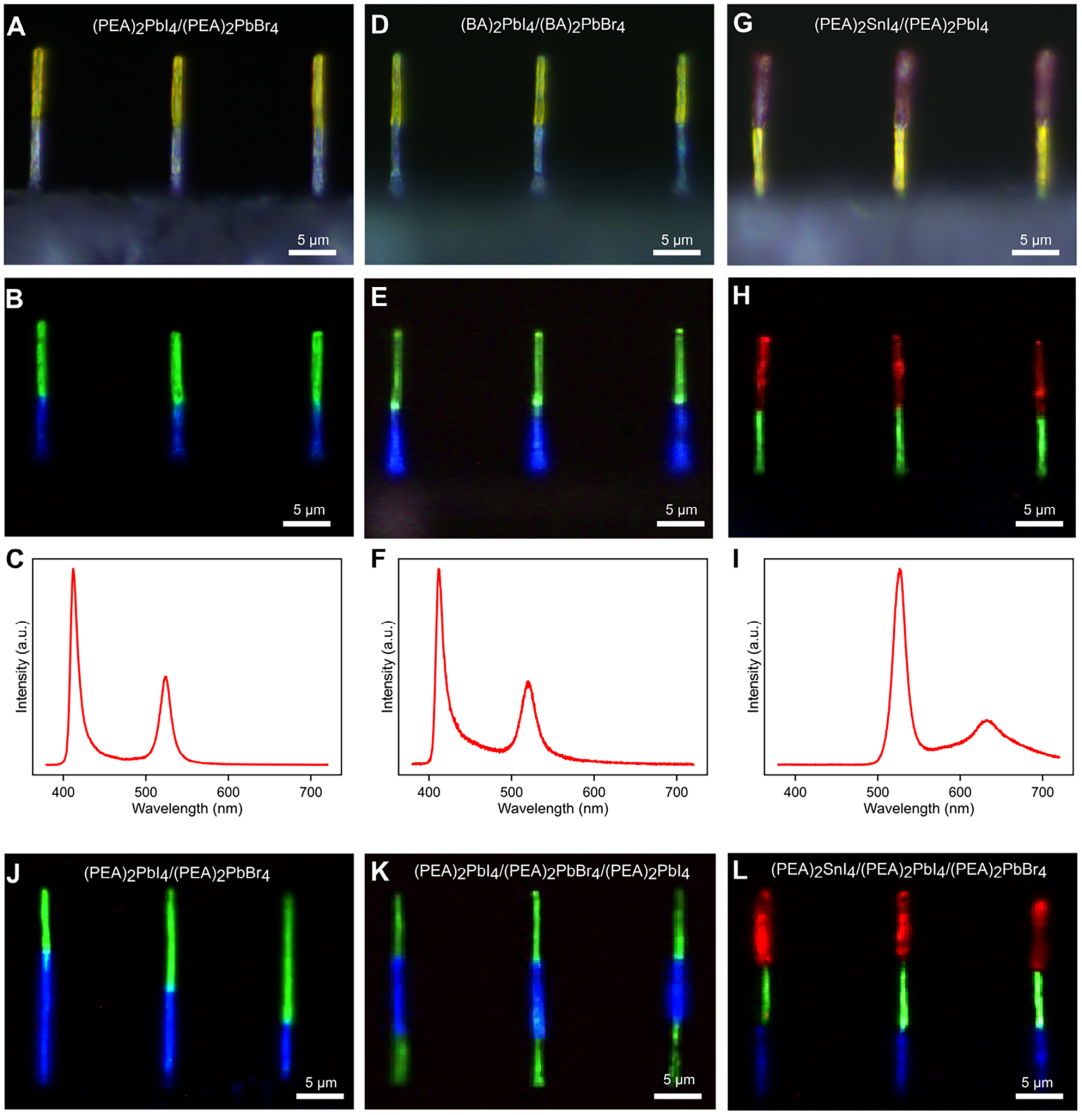
Library of layered halide perovskite nanowire heterostructures fabricated by femtoliter meniscus‐guided printing. (A–C) Halide substitution in (PEA)_2_PbI_4_/(PEA)_2_PbBr_4_ heterostructures, showing dual‐color PL emission at 527 nm (iodide, green) and 411 nm (bromide, blue), confirming sharp compositional interfaces. (D–F) Ligand substitution in (BA)_2_PbI_4_/(BA)_2_PbBr_4_ heterostructures yields dual‐color emission (522, 410 nm) with a slight spectral shift relative to PEA analogues, reflecting organic spacer effects on bandgap tuning. (G–I) Cation substitution in (PEA)_2_SnI_4_/(PEA)_2_PbI_4_ heterostructures produces distinct red (630 nm) and green (527 nm) emissions, demonstrating the incorporation of Sn^2^^+^ segments. (J) PL images of (PEA)_2_PbI_4_/(PEA)_2_PbBr_4_ heterostructures with controlled length ratios (2:1, 1:1, 1:2), highlighting sub‐micrometer precision in segment engineering. (K) Symmetric triple heterostructure (PEA)_2_PbI_4_/(PEA)_2_PbBr_4_/(PEA)_2_PbI_4_. (L)Ternary triple heterostructure (PEA)_2_SnI_4_/(PEA)_2_PbI_4_/(PEA)_2_PbBr_4_.

We next leveraged the programmability of femtoliter meniscus‐guided nanoprinting to build a comprehensive library of layered halide perovskite nanowire heterostructures with tailored compositions and architectures (Figure 3). This library encompasses compositional tuning across halides, organic ligands, and metal cations, together with precise structural control over segment ratios and multi‐junction configurations. We first demonstrated halide substitution by fabricating (PEA)_2_PbI_4_/(PEA)_2_PbBr_4_ nanowire heterostructures (Figure 3A), which exhibited dual‐color emission under UV excitation: green emission at 527 nm for the iodide segment and blue emission at 411 nm for the bromide counterpart (Figure 3B,C). The distinct spectral separation confirms the successful integration of two photonic domains within a single nanowire. Substituting the PEA^+^ ligand with the shorter BA^+^ ligand produced (BA)_2_PbI_4_/(BA)_2_PbBr_4_ nanowire heterostructures (Figure 3D), which also displayed dual‐color emission (522 nm for iodide, 410 nm for bromide) with a slight spectral shift relative to the PEA‐based analogues (Figure 3E,F), reflecting the influence of organic spacer size on the optical bandgap. Furthermore, replacing Pb^2^^+^ with Sn^2^^+^ yielded (PEA)_2_SnI_4_ nanowires and enabled the fabrication of (PEA)_2_SnI_4_/(PEA)_2_PbI_4_ nanowire heterostructures (Figure 3G), which exhibit distinct red (630 nm) and green (527 nm) emissions (Figure 3H,I). We note that the heterojunction region remains structurally continuous, and the slight PL reduction at the interface likely arises from localized non‐radiative centers rather than any physical gap. These examples establish femtoliter meniscus‐guided nanoprinting as a general route to compositionally programmable layered perovskite nanowire heterostructures.

Structural programmability was further demonstrated by precise control of segment ratios. In (PEA)_2_PbI_4_/(PEA)_2_PbBr_4_ nanowire heterostructures, the relative segment lengths could be tuned at will while maintaining a constant overall nanowire length (Figure 3J). Beyond binary systems, a symmetric triple heterostructure (PEA)_2_PbI_4_/(PEA)_2_PbBr_4_/(PEA)_2_PbI_4_ was fabricated (Figure 3K). Introducing a third composition further enabled a ternary architecture, (PEA)_2_PbI_4_/(PEA)_2_PbBr_4_/(PEA)_2_SnI_4_ (Figure 3L). These multi‐segmented nanowire heterostructures highlight the unique ability of femtoliter meniscus‐guided nanoprinting to seamlessly integrate multiple materials within a single programmable nanostructure.

### 2.4 Ion Diffusion Kinetics of the Heterojunction

**FIGURE 4:**
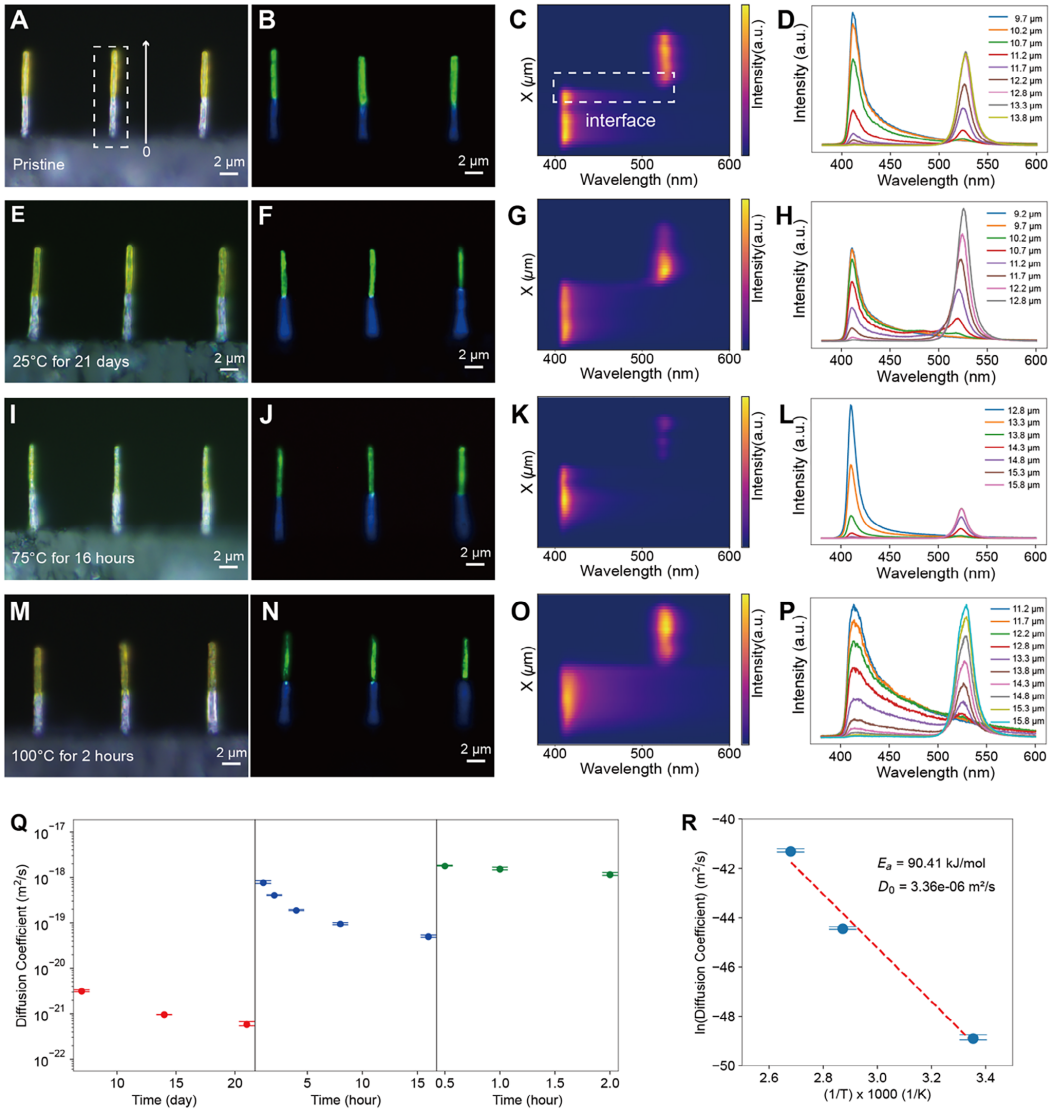
Halide ion interdiffusion in layered perovskite nanowire heterostructures. (A–D) Pristine (PEA)_2_PbI_4_/(PEA)_2_PbBr_4_ heterostructure immediately after printing. (A) Bright‐field optical image. (B) Corresponding real‐color PL image reveals a sharply defined binary interface. (C) Corresponding PL mapping confirms the spatially resolved emission profile. (D) Corresponding interface spectra show an abrupt transition from Br‐rich emission at 411 nm to I‐rich emission at 527 nm, confirming compositional integrity. The legend values represent relative positions along the nanowire axis referenced to the PL scan origin (x = 0). (E–H) After aging at 25°C for 21 days. (E, F) Optical and PL images reveal a faint mixed‐emission zone at the interface. (G,H) PL mapping and spectra indicate only a slight iodide peak shift (527 → 521 nm), confirming minimal interdiffusion under ambient conditions. (I–L) After annealing at 75°C for 16 h. (I,J) Optical and PL images show broadening of the interfacial region. (K,L) PL Mapping and spectra reveal iodide peak shift (527 → 523 nm), evidencing thermally activated interdiffusion. (M–P) After annealing at 100°C for 2 h. (M, N) Pronounced light‐blue band develops at the interface. (O,P) PL mapping and spectra reveal further iodide peak shift (527 → 518 nm), demonstrating accelerated intermixing. (The numbers shown in the legends of panels D, H, L, and P indicate relative positions (in µm) along the nanowire axis, referenced to the scan origin (x = 0), rather than absolute distances from the substrate interface). (Q) Extracted interdiffusion coefficients at 25 (red), 75 (blue), and 100°C (green), determined from PL‐composition calibration and Boltzmann–Matano analysis. (R) Arrhenius plot of diffusion coefficients. Fitted activation energy confirms vacancy‐mediated halide migration. (For each annealing condition, three independent nanowires were tested across separate fabrication batches; representative single‐wire data are shown here, and reproducibility is confirmed in Figure S14).

For these heterostructures, the interfaces critically determine the structural and electronic stability. EDS mapping (Figure 1F) confirms sharp Br/I segregation in pristine nanowires, while spatially resolved photoluminescence (PL) provides a minimally invasive and quantitative probe of interdiffusion, enabling longitudinal monitoring that destructive methods such as TEM‐EDS or XRD cannot achieve. For pristine (PEA)_2_PbI_4_/(PEA)_2_PbBr_4_ heterostructures measured immediately after fabrication, optical and PL characterization (Figure 4A–D) revealed a sharply defined interface, with an abrupt transition from 411 nm (Br‐rich) to 527 nm (I‐rich). After 21 days at 25°C (Figure 4E–H), the interface exhibited only a faint light‐blue region, accompanied by a slight iodide peak shift (527 → 521 nm), while the bromide peak remained unchanged. The PL mapping showed negligible broadening, confirming minimal interdiffusion under ambient conditions. By contrast, thermal annealing markedly accelerated ion migration. At 75°C for 16 h (Figure 4I–L), the iodide peak shifted to 523 nm, and the interfacial region broadened into a mixed‐emission zone. Annealing at 100°C for 2 h (Figure 4M–P) drove further intermixing, shifting the iodide peak to 518 nm and expanding the interface into a distinct light‐blue band. The systematic evolution of PL images and spectra is summarized in Figures S8–S10.

To quantitatively analyze diffusion kinetics, the heterostructure was modeled as a 1D binary diffusion couple. The diffusion coefficient varies along the nanowire heterojunction due to the concentration gradient at the interface. This transient, 1D diffusion is governed by Fick's second law [40]:

(2) $$\frac{\partial c}{\partial t}=D\left(c\right)\frac{\partial^{2}c}{\partial x^{2}}$$

where *c* is the bromide concentration, *t* is the time, *x* is the position along the nanowire axis, and *D(c)* is the concentration‐dependent diffusion coefficient. To correlate PL emission with local halide composition, a calibration series of (PEA)_2_PbBr_4__x_I_4__(__1__−__x__)_ nanowires (*x* = 0–1) was fabricated. Their PL spectra exhibited a linear dependence of emission energy on Br fraction (Figure S11). This calibration allowed direct conversion of PL line‐scan profiles into quantitative composition maps. The resulting concentration profiles were fitted using a complementary cumulative distribution function (CCDF), and the concentration‐dependent diffusion coefficient *D(c)* was then extracted using the BM method (details are provided in the Supporting Information) [41]. Unlike thin‐film studies where BM analysis is an approximation, vertically aligned nanowires provide an intrinsically 1D transport pathway along the growth axis, enabling a more rigorous determination of *D*(*c*).

The extracted interdiffusion coefficients are shown in Figure 4Q. At 25°C, the diffusion coefficients remain low and show a slight decrease with aging time, consistent with negligible halide transport under ambient conditions. In contrast, annealing at 75°C and 100°C produced progressively larger coefficients, consistent with thermally activated ion migration. The slight apparent decrease in diffusion coefficients at long annealing times arises from both the intrinsic flattening of concentration gradients and the sensitivity of BM analysis to evolving boundary conditions. The diffusion coefficient of the (PEA)_2_PbI_4_/(PEA)_2_PbBr_4_ heterostructure agrees with literature values *D(T)* (∼10^−^^18^ m^2^ s^−^^1^ at 100°C) [32], and is more than two orders of magnitude lower than that of conventional 3D perovskites (∼10^−^^16^ m^2^ s^−^^1^ at 100°C) [42]. This strong suppression of halide intermixing indicates the sharp interface, which is fundamentally rooted in the lattice anisotropy of the layered halide perovskites. Unlike the continuous inorganic network in 3D perovskites, organic spacer layers interrupt continuous out‐of‐plane diffusion pathways and impose a high barrier to cross‐layer hopping, thereby strongly suppressing interdiffusion across the heterointerface [18, 43]. The spacer chemistry of the bulky PEA spacers further intensifies this effect. The large steric hindrance and rigid packing of the aromatic rings increase the effective activation barrier for vacancy‐mediated halide hopping across the organic–inorganic interface [19, 32]. Moreover, our 3D freestanding geometry may minimize extrinsic factors such as lattice strain, which is known to facilitate ion diffusion in substrate‐constrained thin films [44, 45].

By combining spatially resolved PL mapping with Arrhenius analysis, we evaluated the temperature dependence of halide diffusion. The thermally activated process is described by the Arrhenius equation [46]:

(3) $$D\left(T\right)=D_{0}\;exp\left(\frac{-E_{a}}{RT}\right)$$

where *D*_0_ is the pre‐exponential factor, *E_a_* is the activation energy for vacancy‐mediated halide migration, *R* is the universal gas constant (8.314 J mol^−1^ K^−1^), and *T* is the temperature. Fitting the measured diffusion coefficients (Figure 4R) yielded an activation energy of ∼0.93 eV (90 kJ mol^−^^1^), which is substantially higher than the 0.1–0.6 eV typically reported for 3D perovskites [47]. This elevated activation energy suggests a significantly increased barrier for vacancy‐mediated halide migration in our printed nanowire heterostructures. The Arrhenius fit captures the exponential increase of *D* with temperature, supporting the mechanistic assignment and the quantitative validity of the extracted diffusion coefficients in the nanowire geometry.

### 2.5 Stability of Nanowires and Their Heterostructures

**FIGURE 5:**
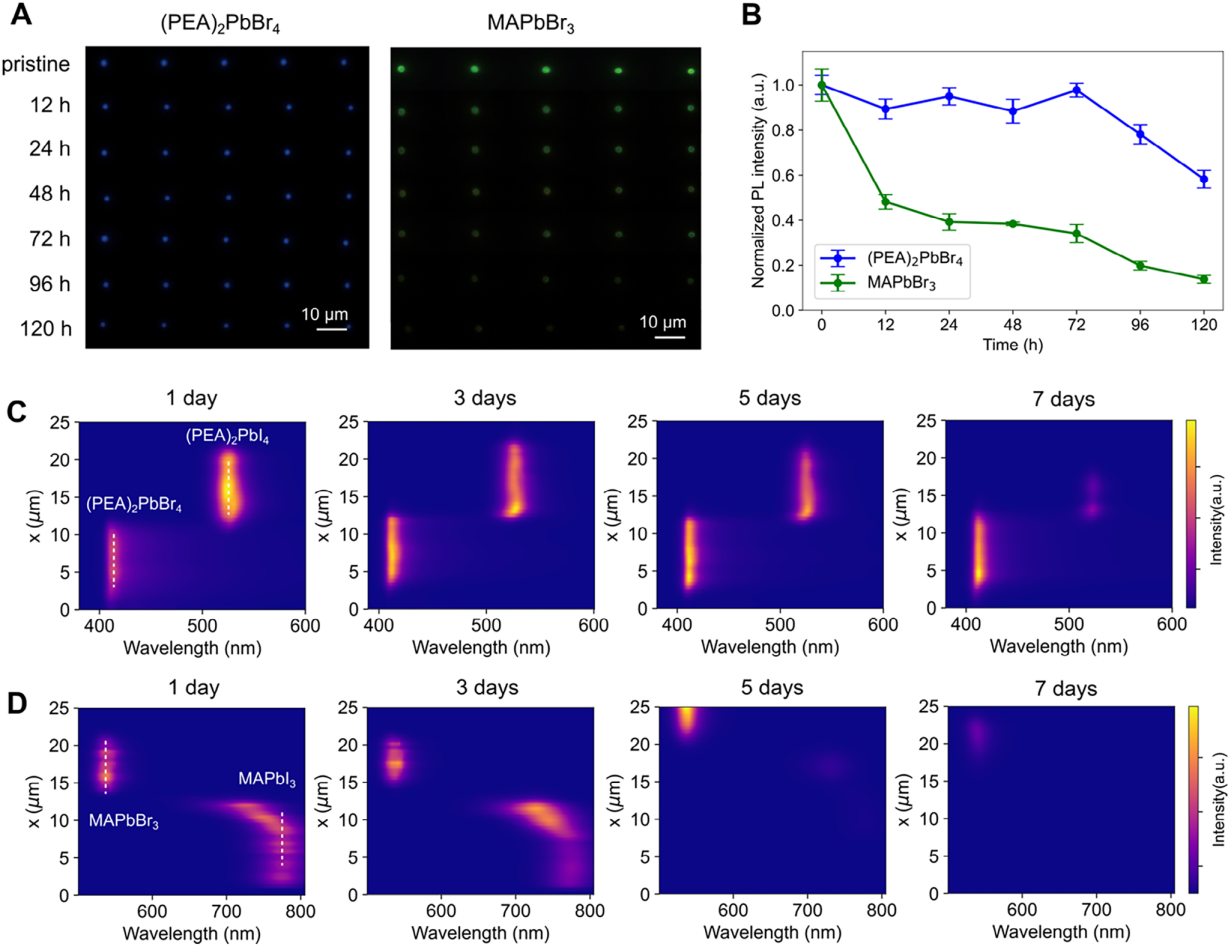
Comparative environmental stability of layered vs. 3D perovskite nanowires and heterostructures. (A) Top‐view real‐color PL images of freestanding layered (PEA)_2_PbBr_4_ nanowire arrays and 3D MAPbBr_3_ nanowire arrays recorded under ambient air (5%–10% RH, room temperature) over 120 h. (B) Corresponding PL intensity–time traces, showing that layered nanowires retain ∼60% of their initial emission after 120 h, while 3D nanowires decay below 10% of their initial emission. (C) PL mapping of (PEA)_2_PbI_4_/(PEA)_2_PbBr_4_ nanowire heterostructures after one week of ambient storage (25°C, 5–10% RH), preserving sharp dual‐color emission and interface integrity. (D) PL mapping of MAPbI_3_/MAPbBr_3_ heterostructures under identical conditions, exhibiting pronounced PL quenching and spectral broadening at the junction due to accelerated halide diffusion.

The environmental stability of 3D‐printed layered perovskite nanowires was systematically benchmarked against their 3D perovskite counterparts under controlled ambient conditions (5%–10% RH, room temperature). Top‐view PL images (Figure 5A) revealed that (PEA)_2_PbBr_4_ nanowires maintained bright and uniform emission throughout 120 h of storage, whereas MAPbBr_3_ nanowires underwent rapid degradation, with their emission nearly extinguished after 96 h. Corresponding quantitative PL intensity–time traces (Figure 5B) reveal that (PEA)_2_PbBr_4_ nanowires preserved ∼60% of their initial emission after 120 h, while MAPbBr_3_ nanowires decayed below 10% over the same period. This contrast is consistent with the layered, quantum‐well–like architecture that hinders water ingress and ion migration, improving ambient stability [48].

We next assessed the stability of heterostructures, as junction integrity is a critical determinant of device reliability. The (PEA)_2_PbI_4_/(PEA)_2_PbBr_4_ heterostructures (Figure 5C) preserved sharp dual‐color emission and narrow junction interfaces after one week, confirming suppressed halide interdiffusion and environmental stability. This behavior is consistent with ion‐blocking van der Waals gaps in layered perovskites [33]. By contrast, MAPbI_3_/MAPbBr_3_ heterostructures exhibited rapid degradation (Figure 5D), showing PL quenching and junction spectral broadening associated with accelerated halide interdiffusion in 3D perovskites. These results highlight the markedly improved environmental stability of layered nanowires and their heterostructures over 3D analogues.

### 2.6 Printed Nanowire Heterojunction Photodetectors

**FIGURE 6:**
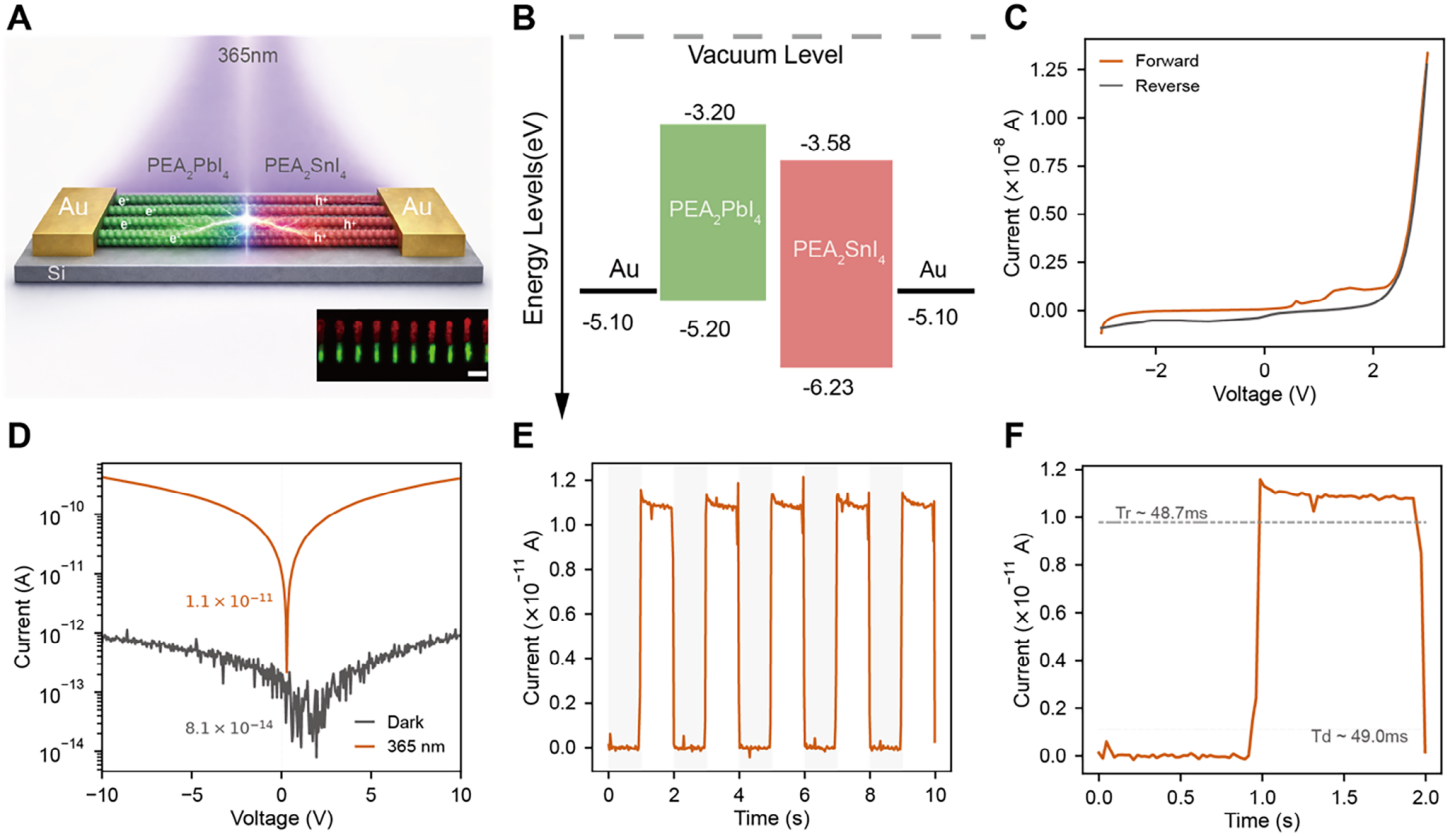
Device‐level demonstration of 3D‐printed nanowire heterostructures. (A) Schematic of the photodetector architecture based on vertically aligned (PEA)_2_PbI_4_/(PEA)_2_SnI_4_ nanowire heterostructures encapsulated with PMMA and integrated between Au electrodes. The inset shows a corresponding PL image of the heterostructure array (scale bar: 50 µm). (B) Energy band diagram illustrating the type‐II alignment between Pb‐ and Sn‐based perovskite segments, establishing a built‐in electric field for carrier separation. (C) Dark I–V characteristics showing diode‐like rectification, confirming the formation of a functional p–n junction. (D) I–V curves under dark and 365 nm UV illumination, revealing a pronounced photocurrent at zero bias, consistent with self‐powered operation. (E) Time‐resolved on–off photoresponse at 0 V, showing stable and reproducible switching over multiple cycles. (F) Temporal response dynamics of the device under pulsed illumination, yielding rise and decay times of ∼49 ms.

At the device level, we fabricated prototype photodetectors by directly integrating 3D‐printed nanowire heterostructure arrays between Au electrodes, followed by PMMA encapsulation for environmental stability (Figure 6A). The expected energy‐level alignments for representative heterojunctions are summarized in Figure S16. (PEA)_2_PbI_4_/(PEA)_2_SnI_4_ is a type‐II alignment, where staggered band edges generate an internal electric field that promotes efficient charge separation. In this configuration, the aligned nanowire heterostructures establish a built‐in p–n junction (Figure 6B). Dark I–V characteristics (Figure 6C) shows clear diode‐like rectification that validates junction formation. For comparison, type‐I aligned heterostructures such as (PEA)_2_PbBr_4_/(PEA)_2_PbI_4_ display nearly ohmic conduction with negligible rectification (Figure S17), highlighting the importance of type‐II alignment for functional device operation.

The (PEA)_2_PbI_4_/(PEA)_2_SnI_4_ nanowire heterostructure photodetectors were evaluated at zero bias, confirming self‐powered operation driven by the built‐in electric field (Figure 6D). Under 365 nm illumination (3.2 mW cm^−^^2^), the 10‐nanowire array (effective illuminated area: 2.5 × 10^−^^5^ cm^2^) yielded a responsivity of R = 1.39 × 10^−^^4^ A W^−^^1^. Assuming shot‐noise‐limited dark‐current noise at 0 V, we calculated a specific detectivity D* = 4.3 × 10^9^ Jones and noise‐equivalent power NEP = 1.16 × 10^−^^1^ ^2^ W Hz^−^^1^/^2^ (derivations in the Supporting Information). The device exhibited stable on‐off switching (<1% drift over 28 cycles) and transient rise/decay times of ≈49 ms. While R and D* are modest compared to biased benchmarks (Table S1), our proof‐of‐concept device achieves ultralow dark current and true zero‐bias operation in a programmable heterojunction architecture. Further improvements in crystal quality and contact engineering are expected to narrow the performance gap with conventional devices. These results establish femtoliter meniscus‐guided nanoprinting as a direct route to heterostructure devices that integrate rectifying diode behavior with fast, self‐powered photodetection—key attributes for next‐generation, low‐energy, and high‐speed optoelectronic applications.

## 3 Conclusion

In summary, the femtoliter meniscus‐guided 3D nanoprinting provides a general and programmable approach for constructing layered halide perovskite nanowire heterostructures with deterministic control over geometry, multi‐segment composition, and sharp interfaces. By incorporating the deterministic crystallization of layered perovskites into the meniscus‐guided process, we overcome the interfacial degradation common in 3D systems and establish a robust platform for the 3D printing of functional nanodevices. This strategy goes beyond conventional thin‐film or lithography‐based methods by enabling direct, freeform integration of functional heterostructures at the nanoscale. Through spatially resolved photoluminescence mapping coupled with BM diffusion analysis, we quantify halide interdiffusion across heterointerfaces and reveal diffusion coefficients more than two orders of magnitude lower than those of 3D counterparts. These results provide a rigorous nanoscale framework that explains the superior environmental and interfacial stability of layered systems beyond the qualitative insights available from thin‐film studies. At the device level, self‐powered nanowire photodetectors exhibit rectifying behavior, pronounced zero‐bias photocurrent, and sub‐50 ms response times, directly linking nanoscale structural programmability to functional performance. These advances elevate meniscus‐guided nanoprinting from a crystallization technique to a versatile manufacturing platform for durable, programmable optoelectronic nanodevices.

## 4 Experimental Section

### 4.1 Materials

Phenethylammonium iodide (PEAI, 99.9%), phenethylammonium bromide (PEABr, 99.9%), n‐butylammonium iodide (BAI, 99.9%), n‐butylammonium bromide (BABr, 99.9%), methylammonium iodide (MAI, 99.9%), and methylammonium bromide (MABr, 99.9%) were purchased from Greatcell Solar Materials. Lead iodide (PbI_2_, 99.99%) was obtained from TCI. Lead (II) bromide (PbBr_2_, 99.99%), tin (II) iodide (SnI_2_, 99.99%), N,N‐dimethylformamide (DMF, 99.99%), dimethyl sulfoxide (DMSO, 99.5%), chlorobenzene (99.9%), ethanol, acetone, and isopropanol were procured from Sigma–Aldrich. All chemicals were used as received without further purification.

### 4.2 Ink Preparation

The (PEA)_2_PbI_4_ precursor solution (1.25 m) was prepared by dissolving PEAI and PbI_2_ in a solvent mixture of DMF and DMSO (volume ratio 4:1). Similarly, the (PEA)_2_PbBr_4_ precursor solution (0.1 m) was prepared by dissolving PEABr and PbBr_2_ in DMF: DMSO (4:1). The ink concentration was referenced to the B‐site metal halide precursor concentration (i.e., the molarity of PbX_2_ or SnX_2_), and the corresponding organic halide (e.g., PEAI or PEABr) was added in the stoichiometric ratio. Specifically, the inks used for printing were 1.25 m (PEA)_2_PbI_4_, prepared from 1.25 m PbI_2_ and 2.50 m PEAI, and 0.10 m (PEA)_2_PbBr_4_, prepared from 0.10 m PbBr_2_ and 0.20 m PEABr, unless otherwise specified. For BA‐based compositions, (BA)_2_PbI_4_ and (BA)_2_PbBr_4_ precursor solutions were prepared by dissolving BAI and PbI_2_, or BABr and PbBr_2_, respectively, in the same solvent mixture. The (PEA)_2_SnI_4_ solution was prepared by dissolving PEAI and SnI_2_ in DMF: DMSO (4:1). For 3D perovskites, MAPbI_3_ (0.5 m) and MAPbBr_3_ (0.2 m) precursor solutions were prepared using MAI + PbI_2_ or MABr + PbBr_2_, respectively, in the same solvent mixture. All solutions were stirred at 65°C for 2 h in an argon‐filled glovebox. Mixed‐halide perovskites (PEA)_2_PbI_4__(__1__−__x__)_Br_4__x_ (x = 0.25, 0.5, 0.75; 0.5 m) were prepared by dissolving PEAI, PEABr, PbI_2_, and PbBr_2_ in stoichiometric ratios in DMF: DMSO (4:1).

### 4.3 Pipette Preparation

Borosilicate nanopipettes with embedded filaments were fabricated using a Flaming/Brown P‐97 micropipette puller (Sutter Instrument). The pulling parameters were optimized to produce pipettes with aperture diameters of approximately 1 µm (Figure S2). Pipette morphology was characterized via field emission scanning electron microscopy (FE‐SEM, Hitachi S‐4800).

### 4.4 Substrate Preparation

Si/SiO_2_ substrates were sequentially cleaned using deionized water, acetone, ethanol, and isopropanol, followed by nitrogen drying. The substrates were then subjected to UV–ozone treatment for 10 min to remove organic contaminants and enhance surface energy prior to use in printing.

### 4.5 3D Printing

3D nanoprinting was carried out using a custom‐built printer housed within an environmental chamber. The system comprised a printing head and a build platform, each mounted on motorized linear stages (Kohzu) with sub‐micrometer resolution and centimeter travel, controlled via a Python‐based interface. A 1 µm aperture glass micropipette filled with precursor solution was positioned by a three‐axis stage (x, y, z), while the silicon substrate was placed on a five‐axis stage (x, y, z, θx, θy). This configuration enabled continuous, high‐precision manipulation of the femtoliter meniscus formed at the pipette aperture. The substrate temperature was regulated by a thermoelectric cooling chip, monitored with a MAX6675 K‐type thermocouple, and controlled through an Arduino‐based PID feedback system. Humidity was maintained at 5%–10% RH using an HYT221 sensor and a PID‐controlled solenoid valve for mixing compressed dry and wet air. Real‐time monitoring of the meniscus and growth front was achieved with a long‐working‐distance 50× objective lens (Mitutoyo, depth of field = 0.9 µm), side‐mounted LED illumination, and a CCD camera (Blackfly, Teledyne FLIR).

In this study, typical substrates were ∼1 × 1 cm^2^ in size. At a stage speed of ∼2 µm s^−1^, the fabrication of a 10 µm + 10 µm heterostructure required ∼1 min (including pipette exchange), while arrays of 5 × 5 nanowires were completed within ∼5 min (see Supporting Information, Movies S1 and S2, for representative printing sequences). The entire printing process was normally ≤ 30 min, much shorter than the timescale for measurable halide diffusion.

### 4.6 Optical and Photoluminescence Imaging

Optical and real‐color PL images were obtained using a Leica DM2500 LED upright fluorescence microscope. Side‐view imaging was achieved by tilting the sample stage. Excitation was provided by an external light source (Leica EL6000), and a filter cube (Leica A11513873) containing a 340–380 nm bandpass filter (excitation), 400 nm dichroic mirror, and 425 nm long‐pass emission filter was used.

### 4.7 SEM and EDS

Backscattered and high‐resolution SEM images were obtained using a Hitachi S‐4800 field emission scanning electron microscope (FEG SEM) at 5 kV. Elemental composition was analyzed via energy‐dispersive X‐ray spectroscopy (EDS) at 20 kV using the same system.

### 4.8 Transmission Electron Microscopy

The crystallinity of the 3D‐printed structures at the single‐entity level was examined using transmission electron microscopy (TEM) (FEI Tecnai G2 and Thermo Scientific Talos F200X), both operating at an accelerating voltage of 200 kV. TEM samples were prepared by directly fabricating the 3D‐printed structures onto a TEM grid (Ted Pella, lacey carbon type‐A support film, 300 mesh, copper).

### 4.9 Micro‐PL Spectroscopy and PL Mapping

To minimize extrinsic degradation, all samples were stored in an Ar‐filled glovebox with an oxygen concentration of 0.5–1 ppm and a water content of ∼0.02 ppm (measured by Vigor sensors). Micro‐PL spectra and PL intensity maps were collected using a spectral scanning system (MetaTest ScanPro Advance) with 365 nm excitation. The beam was focused through a 100× objective (spot size ∼1 µm) and attenuated with neutral density filters. All measurements were performed under ambient conditions. For PL line‐scan measurements, the spatial coordinate was defined with x = 0 at the scan origin (i.e., the starting point of the line‐scan), rather than the substrate/nanowire interface. This convention was adopted because the optical microscope cannot precisely resolve the physical contact point at sub‐micrometer accuracy. Defining the origin at the scan start ensured that the entire nanowire was consistently captured across all measurements. The PL line‐scan step size was set to 0.5 µm, yielding 20–40 data points across the interfacial region under all annealing conditions.

### 4.10 Electrical and Optoelectronic Characterization

Current–voltage (I–V) measurements of the printed nanowire devices were performed using a two‐probe station (Lakeshore) equipped with a Keysight B1500A semiconductor parameter analyzer. Bias voltages were swept from −10 to +10 V and back to −10 V to minimize hysteresis. For photodetection measurements, the devices were illuminated with a 365 nm UV LED (intensity ∼3.2 mW cm^−^^2^), and the photocurrent was recorded under zero bias conditions. Time‐resolved photoresponse was evaluated by periodically modulating the UV illumination and monitoring the current at 0 V bias, from which rise and decay times were extracted.

## Conflicts of Interest

The authors declare no conflicts of interest.

## Supporting information




**Supporting File 1**: adma72731‐sup‐0001‐SuppMat.docx.

**Supporting File 2**: adma72731‐sup‐0002‐MovieS1.mp4.

**Supporting File 3**: adma72731‐sup‐0003‐MovieS2.mp4.

## Data Availability

Research data are not shared.
