# Supplementary material for: Freeform Fabrication of Layered Halide Perovskite Nanowire Heterojunctions
Source: Adv Mater. 2026 Mar 4;38(27):e22768. doi: 10.1002/adma.202522768 (PMC13173397; doi:10.1002/adma.202522768)
Supplement: Supplementary file 1 — Supporting File 1: adma72731‐sup‐0001‐SuppMat.docx. [file ADMA-38-e22768-s003.docx]

**Supporting Information**

**Freeform Fabrication of Layered Halide Perovskite Nanowire Heterojunctions**

*Sixi Cao^1^, Yu Liu^1^, Zhoufei Gan^1^, Shiqi Hu^1^, Jihyuk Yang^1^, Zhuoran Wang^1^, Mingjian Yuan^2^, Wen-Di Li^1^, Ji Tae Kim^3*^*

S. Cao, Dr. Y. Liu, Z. Gan, Dr. S. Hu, Dr. J. Yang, Z. Wang, Prof. W.-D. Li

^1^Department of Mechanical Engineering, The University of Hong Kong, Hong Kong, China

Prof. M. Yuan

^2^State Key Laboratory of Advanced Chemical Power Sources, Key Laboratory of Advanced Energy Materials Chemistry (Ministry of Education), Frontiers Science Center for New Organic Matter, College of Chemistry, Nankai University, Tianjin, China

Prof. J.T. Kim

^3^Department of Mechanical Engineering, Korea Advanced Institute of Science and Technology (KAIST), Science Town, Daejeon 34141, Republic of Korea

*Email: [jitae.kim@kaist.ac.kr](mailto:jitae.kim@kaist.ac.kr)

**1 The Mechanism of Meniscus-Guided Nanoprinting**

The meniscus-guided nanoprinting process is governed by the coupled effects of interfacial force balance and evaporation-induced crystallization.

**1.1 Interfacial Force Balance**

The stability of the liquid meniscus bridging the micropipette tip and the growing nanowire is determined by the balance of interfacial tensions at the three-phase contact line, described by the classical Neumann quadrilateral relation:

$\varphi_{0}=arccos\left( \frac{\gamma_{L}^{2}+\gamma_{S}^{2}-\gamma_{SL}^{2}}{2\gamma_{L}\gamma_{S}} \right)$...(1)

where *φ_0_* is the equilibrium meniscus angle at the contact line, *γ_L_* and *γ_S_* are the surface energies of the precursor liquid and the growing solid, respectively, and *γ_LS_* is the interfacial energy between them. During printing process, the instantaneous contact angle *φ* may fluctuate, but growth remains stable as long as *φ* stays within an allowable deviation from *φ_0_*. When this deviation exceeds the pinning limit, Rayleigh–Plateau instability occurs, leading to meniscus rupture and interrupted growth.

**1.2 Evaporation–Crystallization Balance**

Solvent evaporation at the meniscus is the key driver that couples molecular crystallization to the 3D printing process. Evaporative loss increases local solute concentration, thereby raising the chemical potential and inducing supersaturation relative to the equilibrium solubility at the printing temperature. Crystallization then initiates at the meniscus front. The steady-state growth condition can be expressed as a mass balance:

$\dot{m}=\rho\pi r^{2}v$...(2)

where $\dot{m}$ is the mass flow rate of crystallized solute determined by the solvent evaporation rate, *ρ* is the density of the solid phase, *r* is the nanowire radius, and *v* is the pulling speed. This expression predicts that the nanowire diameter decreases with increasing pulling speed, consistent with the experimental scaling shown in Figures 2C and 2F of the main text.

**
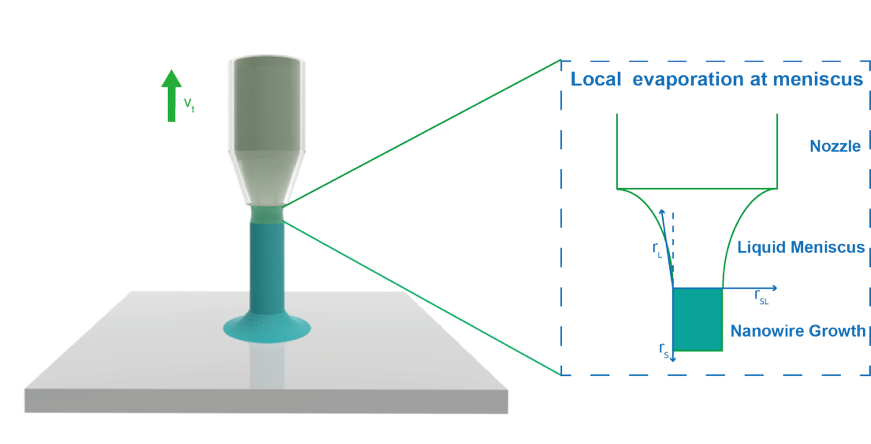
**

**Figure S1 | Mechanistic framework of meniscus-guided nanoprinting.** Stable printing requires simultaneous satisfaction of interfacial force balance at the meniscus and mass balance between evaporation-driven crystallization and pulling speed. These coupled constraints define the operational window for continuous and uniform nanowire growth.

These two criteria together define the operational printing window (Figures 2A–B, 2D–E). At low pulling speeds, excess liquid accumulates in the meniscus, causing uncontrolled lateral crystallization. At excessively high speeds, the meniscus ruptures before crystallization can keep pace. Increasing substrate temperature accelerates evaporation, thereby raising the maximum sustainable pulling speed. However, temperatures above ~70 °C promote uncontrolled supersaturation and tip clogging, as discussed in the main text. Continuous and uniform nanowire printing is achieved only when both criteria are satisfied: the meniscus angle remains within its stable range and the balance between evaporation-driven crystallization and pulling speed is maintained. This mechanistic framework explains the observed dependence of nanowire morphology on pulling speed, precursor concentration, and temperature, and provides a rational basis for optimizing printing parameters.

### 1.3 3D Nanoprinting Setup

The nanoprinting system was custom-built and operated inside an environmental chamber to ensure reproducibility. The setup integrated **the following modules**:

- **Printing head and build platform:** motorized linear stages (Kohzu) with sub-micrometer resolution and centimeter travel, controlled via a Python interface.
- **Nozzle:** glass micropipettes with apertures ranging from 0.8–2 µm, fabricated by laser-assisted pulling. The pipette was mounted on a three-axis stage (*x, y, z*), while the substrate was mounted on a five-axis stage (*x, y, z, θx, θy*).
- **Environmental control:** humidity was stabilized at 5–10% RH by a PID-controlled solenoid valve mixing dry and wet compressed air, monitored using an HYT221 sensor. Substrate temperature was regulated via a thermoelectric cooling chip, controlled by an Arduino-based PID loop with feedback from a MAX6675 K-type thermocouple.
- **Imaging and monitoring:** a long-working-distance 50× objective (Mitutoyo, DOF = 0.9 µm) with side-mounted LED illumination, coupled to a CCD camera (Blackfly, Teledyne FLIR), provided real-time visualization of the meniscus and growth front.


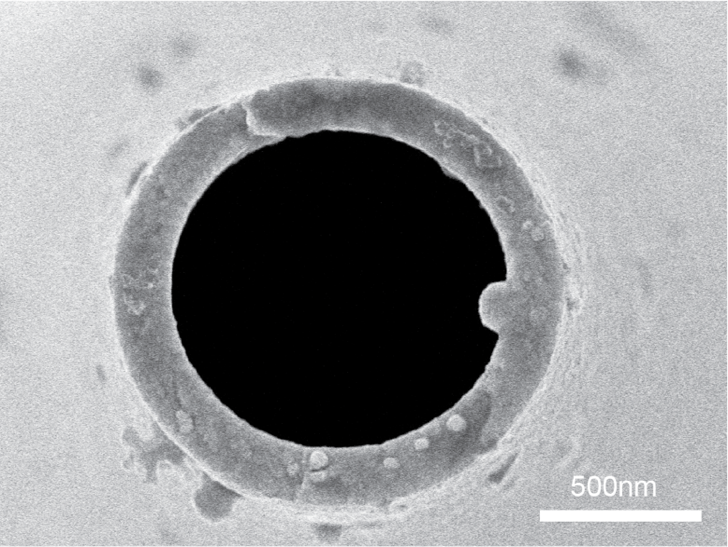


**Figure S2 | FE-SEM image of the glass nanopipette used for 3D printing. (Scale bar: 500 nm)**

### Meniscus Confinement and Volume Estimation. The meniscus volume is estimated by a spherical cap approximation: where *R = d/2* is the radius determined by the nozzle aperture *d*, and *h* is the cap height, set by the contact angle *θ* at the substrate. For a nozzle aperture of 1 µm and contact angle *θ* ≈ 30–40°, the calculated volume is in the femtoliter range (~10⁻¹⁵ L).

$V= \frac{\pi h^{2}}{3}(3R-h)$...(3)

**Surface Tension in Meniscus-Guided Growth**. Although the surface tension of the precursor inks was not directly measured in this study, the experimental trends clearly reflect its influence on meniscus stability. Inks with insufficient surface tension tended to spread and collapse, while overly strong confinement hindered smooth ink supply and led to irregular growth. Stable freestanding nanowires were consistently obtained with optimized solvent formulations, which we infer to correspond to an intermediate surface-tension regime **consistent with values reported for comparable perovskite ink systems in the literature (typically on the order of tens of mN m⁻¹). Nozzle diameter and surface tension together define** the operational window for reliable 3D nanowire printing.

**Effect of nozzle size:** Small apertures (0.8–1.0 µm) provide stronger confinement and yield uniform solid nanowires with stable freestanding growth. Larger apertures (>2.0 µm) destabilize the meniscus, accelerate edge crystallization, and promote hollow tubular morphologies (Figure S3).

**
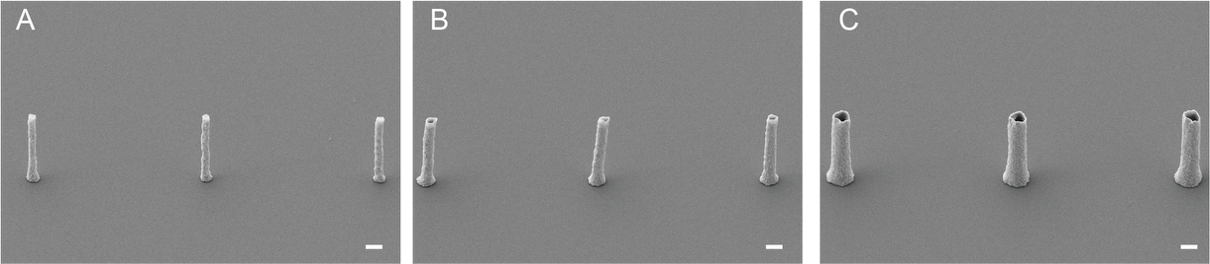
**

**Figure S3 | Effect of nozzle diameter on printed nanowire morphology.** Representative FE-SEM images of (PEA)₂PbI₄ nanowires printed using different nozzle diameters: (A) ~1 µm, yielding uniform solid nanowires with stable meniscus confinement; (B) ~1.5 µm, producing thicker nanowires with partial surface crystallization; and (C) > 2 µm, resulting in hollow tubular morphologies due to accelerated edge crystallization and shell-first growth. Scale bars: 2 µm.

### Clogging Issue. Another important consideration is clogging caused by premature solvent evaporation inside the nozzle. At high precursor concentrations (≥ 1.5 M) or elevated substrate temperatures (> 80 °C), uncontrolled supersaturation at the pipette tip can trigger in-nozzle crystallization and clogging. To avoid this, stable meniscus-guided growth was carried out with precursor concentrations ≤ 1.25 M and substrate temperatures ≤ 70 °C, which effectively suppressed clogging while maintaining reproducible nanowire printing*.*


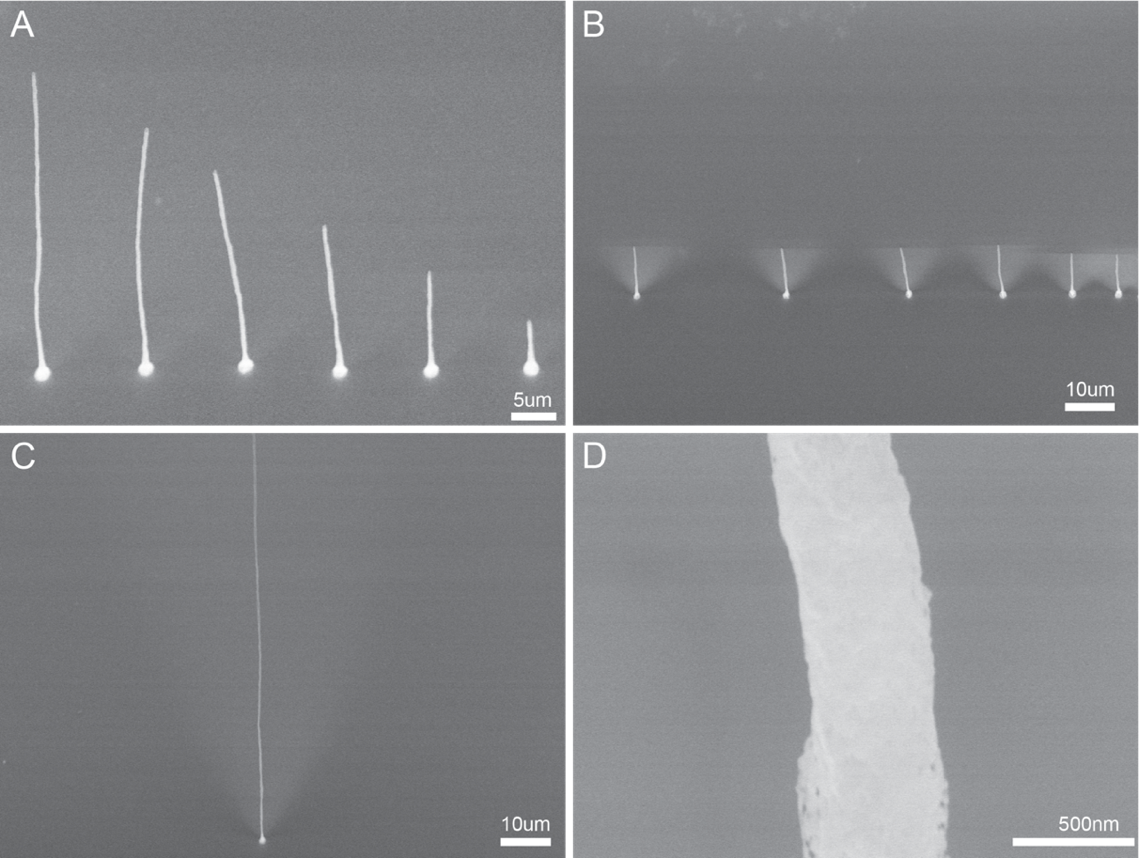


**Figure S4 | Morphological control of 3D-printed nanowire arrays and individual structures.** **(A)** Nanowire arrays with varying heights: 30  µm, 25  µm, 20  µm, 15  µm, and 10  µm (from left to right). **(B)** Nanowire arrays with varying pitch distances: 30  µm, 25  µm, 20  µm, 15  µm, and 10  µm (from left to right). **(C)** A single freestanding nanowire with a high aspect ratio ( > 200 ). **(D)** High-resolution FE-SEM image of the nanowire shown in (C), highlighting its well-defined structure.


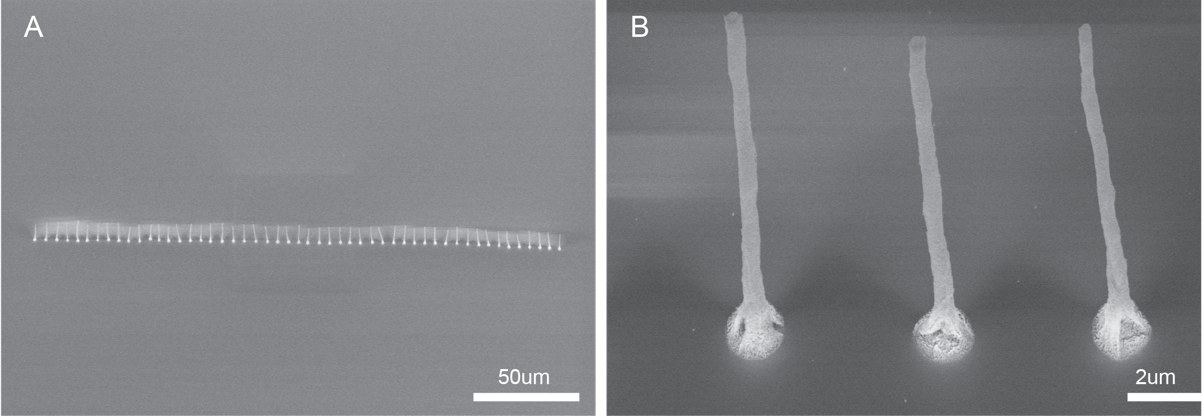


**Figure S5 | Structural analysis of printed nanowire arrays. (A)** FE-SEM image of a scalable nanowire array comprising 50 vertically aligned nanowires with a pitch distance of 5 µm, demonstrating the high-throughput and reproducible capability of the meniscus-guided nanoprinting strategy. **(B)** High-resolution FE-SEM image of representative individual nanowires, showing uniform diameters and smooth sidewalls, confirming the structural fidelity of the printing process.


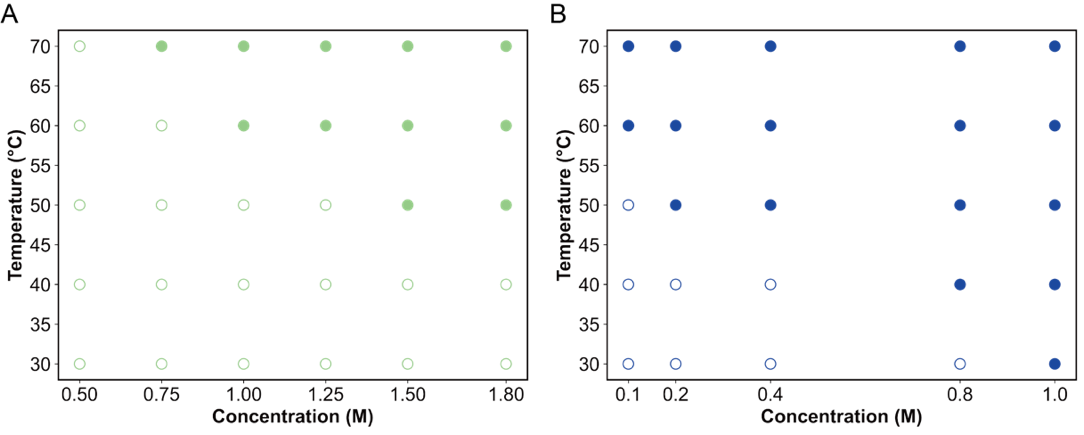


**Figure S6 | Printable windows for layered halide perovskite nanowires using DMSO as the solvent. (A)** Processing window for (PEA)₂PbI₄ nanowires. **(B)** Processing window for (PEA)₂PbBr₄ nanowires.

**
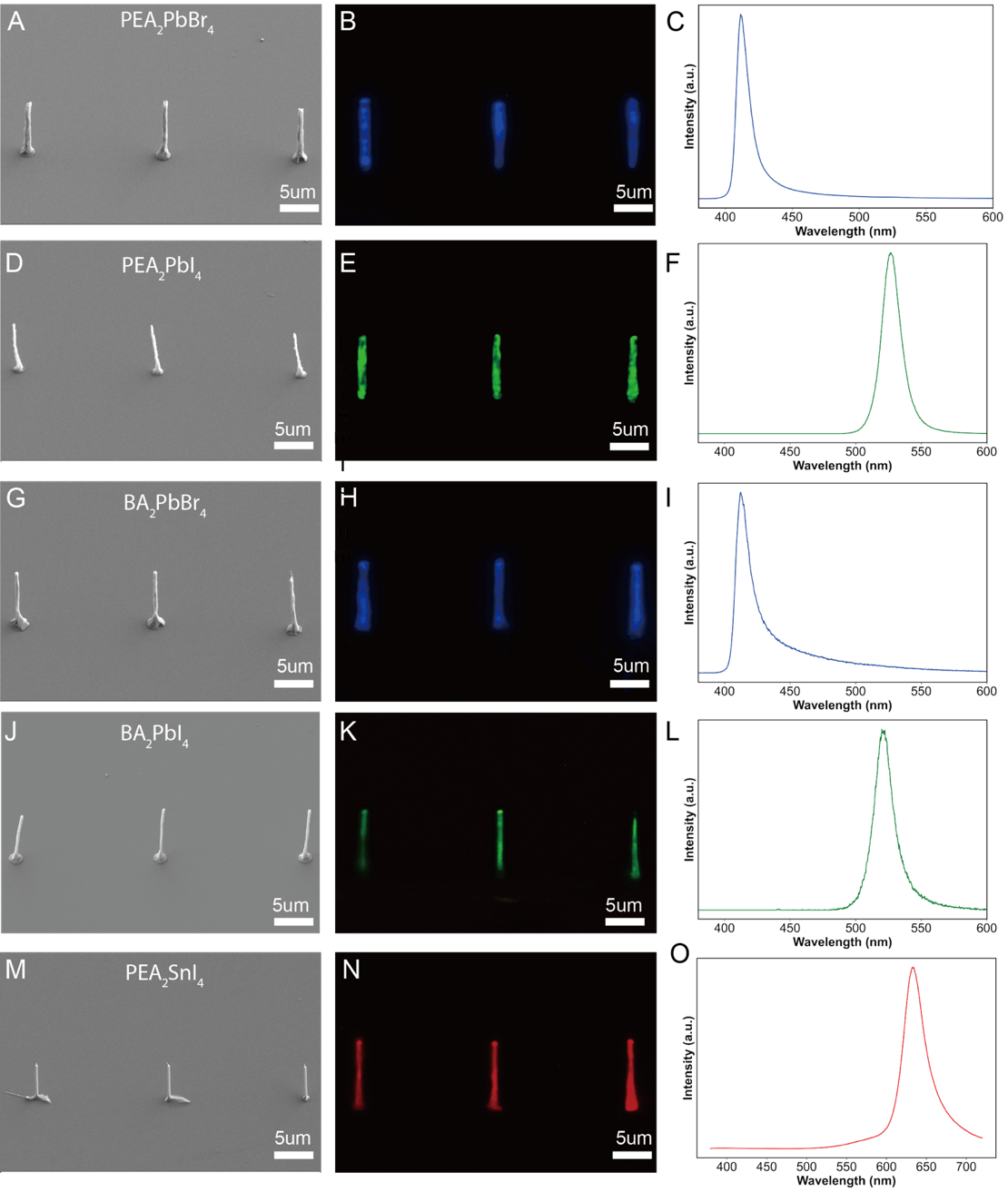
**

**Figure S7 | Structural and optical characterization of single segment layered halide perovskite nanowires.** From top to bottom: **(A–C)** (PEA)₂PbBr₄, **(D–F)** (PEA)₂PbI₄, **(G–I)** (BA)₂PbBr₄, **(J–L)** (BA)₂PbI₄, **(M–O)** (PEA)₂SnI₄. For each set, the panels from left to right show: SEM image, PL image, and PL spectrum.

**2 Boltzmann–Matano (BM) Analysis for Interdiffusion Process**

Boltzmann–Matano (BM) analysis was applied to quantify concentration-dependent halide interdiffusion across nanowire heterojunctions. The procedure consists of four main steps: (i) calibration of PL peak energy to bromide fraction, (ii) acquisition and smoothing of concentration profiles, (iii) CCDF fitting and Matano plane determination, and (iv) extraction of the interdiffusion coefficient *D(c).* Figures S8–11 provide representative datasets and calibration curves, while Figure S12 shows a worked example of the complete analysis workflow including raw PL data, calibration, CCDF fitting, Matano plane placement, and the final *D(c)* extraction.

**
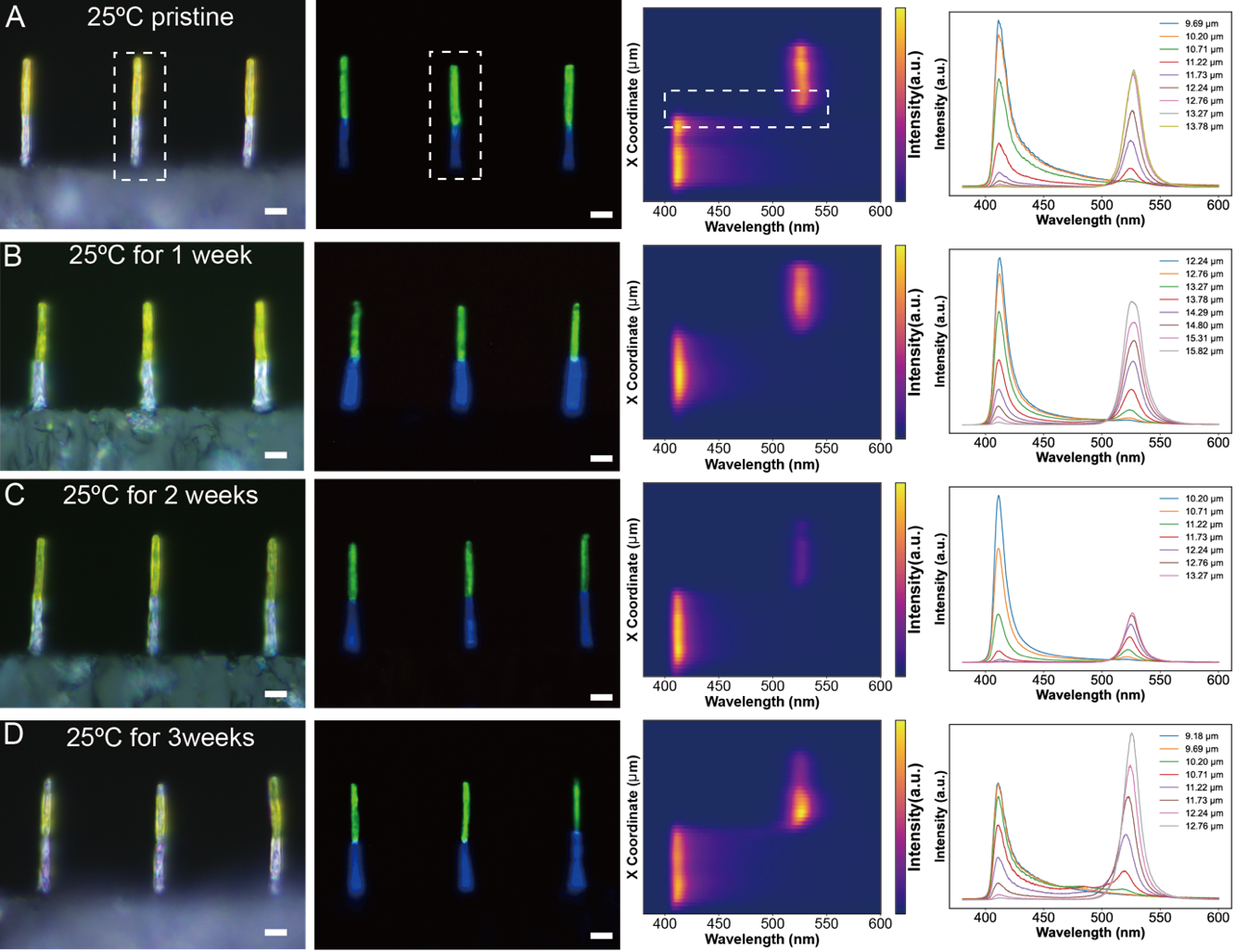
**

**Figure S8** | **Halide ion interdiffusion in (PEA)₂PbI₄/(PEA)₂PbBr₄ nanowire heterojunctions at room temperature (RT, 25 °C). (A–D)** Time-lapse characterization of nanowire heterojunctions at four intervals: pristine (A), after 1 week (B), 2 weeks (C), and 3 weeks (D). Each panel includes, from left to right: bright-field optical microscopy image, real-color PL image, PL intensity mapping, and PL spectrum at the heterojunction interface.


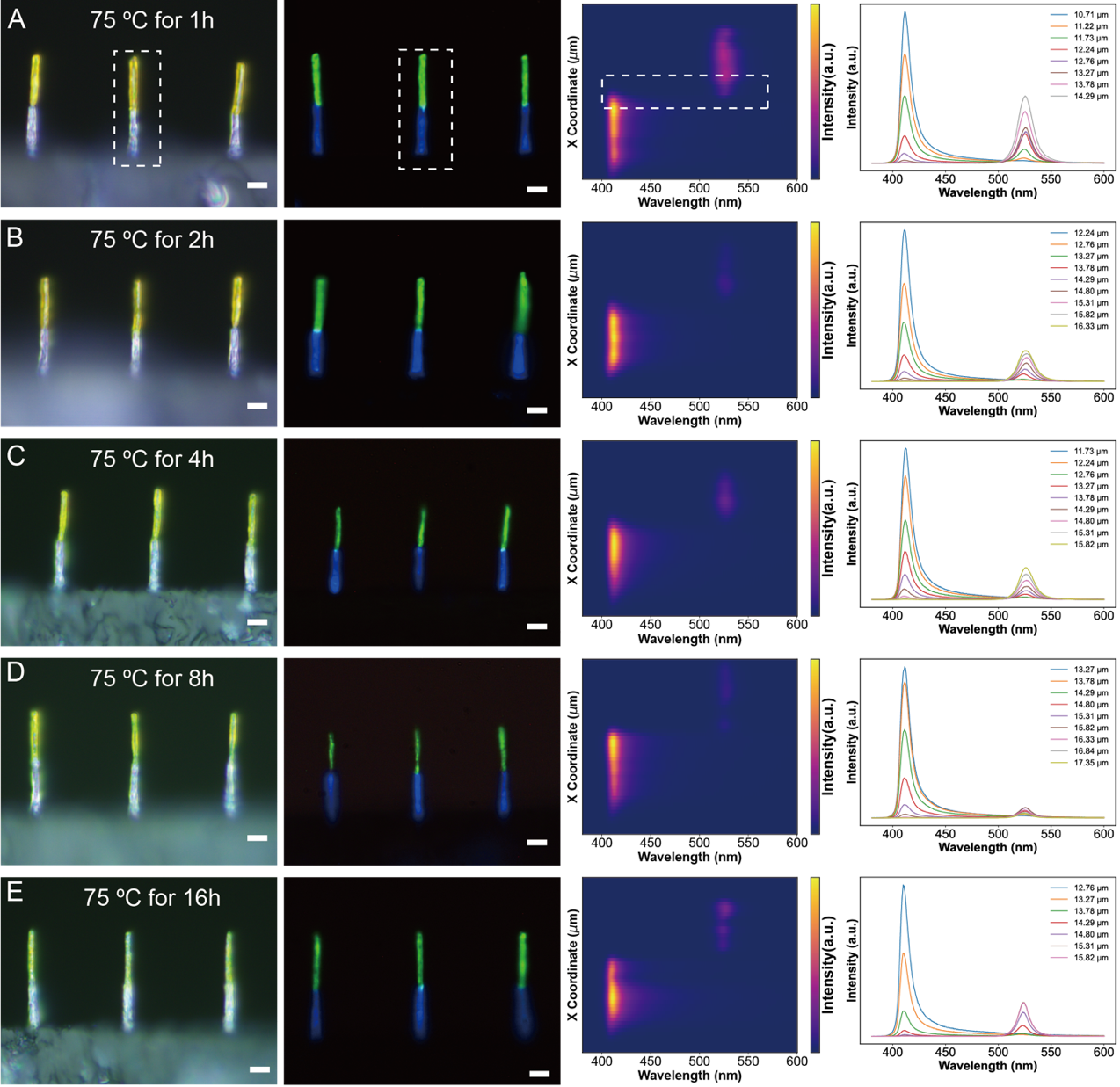


**Figure S9 | Halide ion interdiffusion in (PEA)₂PbI₄/(PEA)₂PbBr₄ nanowire heterojunctions at 75 °C. (A–D)** Time-lapse characterization of nanowire heterojunctions after thermal annealing at 75 °C for (A) 1 h, (B) 2 h, (C) 4 h, and (D) 8 h. Each panel includes, from left to right: bright-field optical microscopy image, real-color PL image, PL intensity mapping, and PL spectrum at the heterojunction interface.


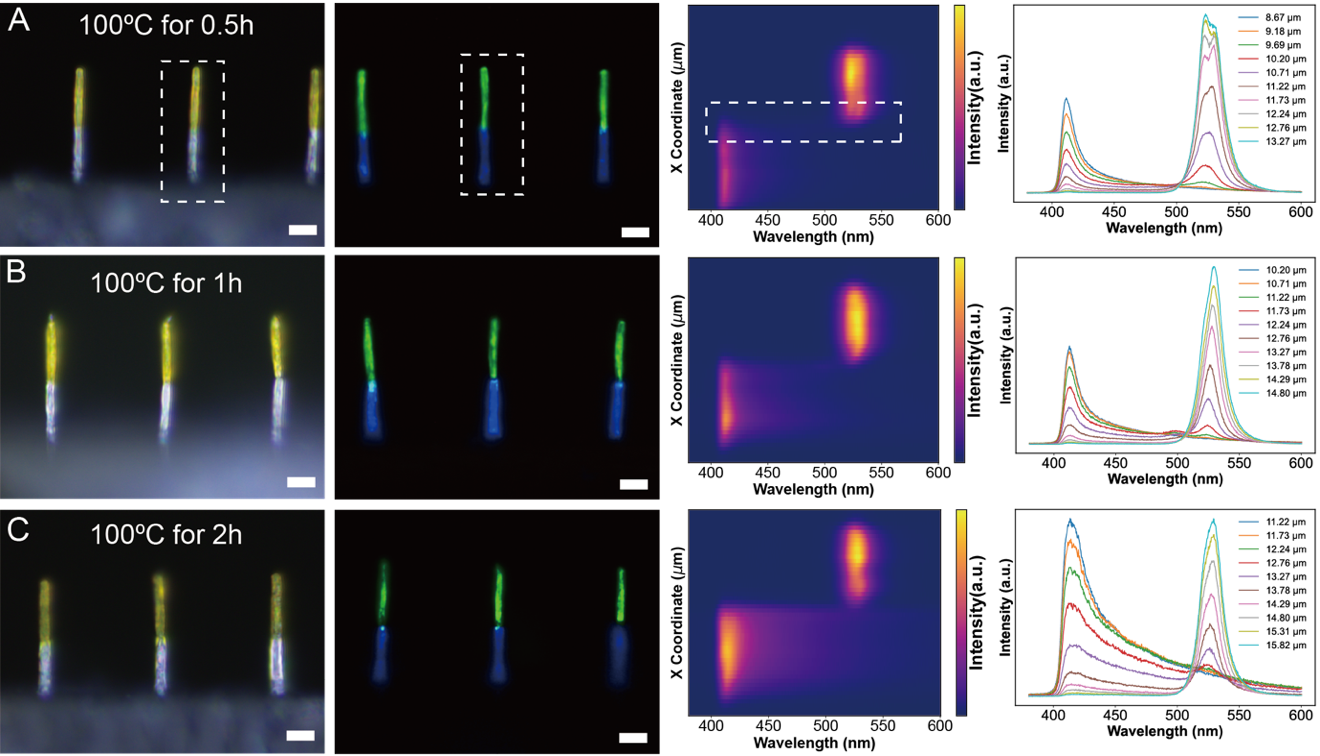


**Figure S10 | Halide ion interdiffusion in (PEA)₂PbI₄/(PEA)₂PbBr₄ nanowire heterojunctions at 100 °C. (A–C)** Time-lapse PL characterization after thermal annealing at 100 °C for (A) 0.5 h, (B) 1 h, and (C) 2 h. Each panel includes, from left to right: real-color PL image, PL intensity mapping, and PL spectrum at the heterojunction interface.

**2.1 1D Diffusion Model and Boundary Conditions**

Each nanowire heterostructure is modeled as a one-dimensional diffusion couple along the wire axis x. The bromide fraction is *c(x,t)* in [0,1], governed by Fick’s second law:

$\frac{\partial c}{\partial t}=D\frac{\partial^{2}c}{\partial x^{2}}$ (4)

where *c* is the bromide concentration, *t* is time, *x* is the position along the nanowire axis, and *D(c)* is the concentration-dependent diffusion coefficient

**Initial condition:** The concentration profile at *t* = 0 was defined as a piecewise distribution with an abrupt but finite-width interface, extracted directly from pristine PL line scans.

**Boundary** **condition**: The analysis domain was restricted to a finite nanowire length centered on the heterojunction. Zero-flux (Neumann) boundary conditions were imposed at both ends, ensuring mass conservation during the diffusion process.

**2.2 PL-to-Composition Calibration**

The bromide fraction X_br_ in the mixed-halide perovskites was estimated directly from the PL emission spectra. First, the peak wavelength λ of each composition was extracted by fitting the normalized PL spectrum with a peak function (e.g., Gaussian) to determine the maximum position. The corresponding photon energy was then calculated according to the relation:

$E= \frac{hc}{}$ (5)

where *h* is Planck’s constant, *c* is the speed of light, and *λ* is the PL peak wavelength. A calibration curve was constructed by plotting the measured photon energies against the nominal bromide composition *X_br_* of the precursor solutions (Figure S11). The data exhibit an approximately linear dependence, which can be expressed as:

$E\left( ev \right)=$*0.66X_br_+2.36*

This empirical relation was subsequently employed to determine the local bromide concentration in nanowire heterostructures based on their PL peak positions. The linearity of the calibration (with *R^2^*=0.99) confirms the reliability of this spectroscopic approach for compositional mapping.

**
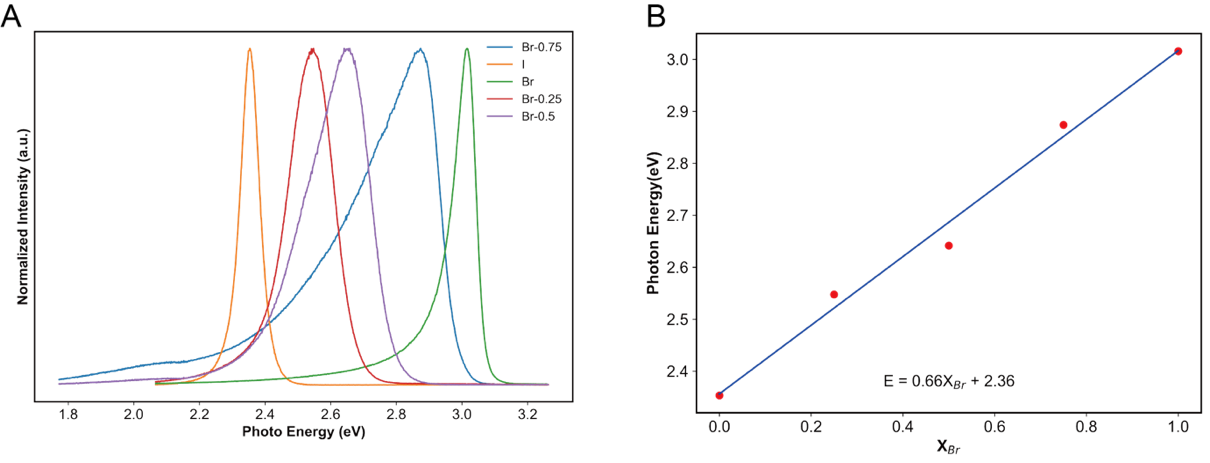
**

**Figure S11 | PL calibration curve for (PEA)₂PbBr₄ₓI₄₍₁₋ₓ₎ halide alloy perovskite nanowires. (A)** Photoluminescence (PL) spectra of printed nanowires with varying halide compositions: X_Br_ = 1, 0.75, 0.5, 0.25, and 0. **(B)** Correlation between PL emission energy and bromide composition. The red scatter plot represents experimentally measured average PL energies, and the blue line indicates a linear fit to the data.

**2.3 Data Pre-Processing and Fitting**

**Sampling :** Line-scan step = 0.5 µm, yielding ~20–40 points across each broadened interface.

**Denoising :** A Savitzky–Golay filter (order 2, adaptive window) is applied to suppress noise while preserving the inflection region.

**CCDF fit :** The smoothed profile is fit to a complementary cumulative distribution function (equivalently, an error-function step):

$c_{fit}\left( x \right)=\frac{c_{L}+c_{R}}{2}-\frac{c_{R}-c_{L}}{2}erf\left( \frac{x-\mu}{\sqrt{2}\sigma} \right)$...(6)

where $\mu$ locates the interface center and $\sigma$ describes the width. Fits yield *R^2^* = 0.90–0.95.

**Matano Plane Determination :** The Matano plane position (*x_M_*) was defined by material conservation:

$\int_{\frac{-L}{2}}^{x_{M}} \left| c\left( x \right)-c_{L} \right|dx=\int_{x_{M}}^{\frac{L}{2}} \left| c\left( x \right)-c_{L} \right|dx$....(7)

All profiles were subsequently shifted to *x = x − x_M_* to ensure symmetry.

**Extraction of Concentration-Dependent Diffusion Coefficient**

The concentration-dependent interdiffusion coefficient *D(c)* was extracted using the Boltzmann–Matano relation:

$D\left( c \right)=-\frac{1}{2t}\left[ \frac{1}{{\partial c}/{\partial x}}\int_{c_{0}}^{c} \left( x-x_{M} \right)dc \right]$…(8)

where *t* is the annealing time, *x* is the spatial coordinate, *x_M_* denotes the position of the Matano plane and $\frac{\partial c}{\partial x}$  is the local slope of the composition profile. This formulation links the spatial broadening of the composition profile to the concentration-dependent diffusivity *D(c),* and is evaluated numerically using the fitted CCDF profile *c(x).*


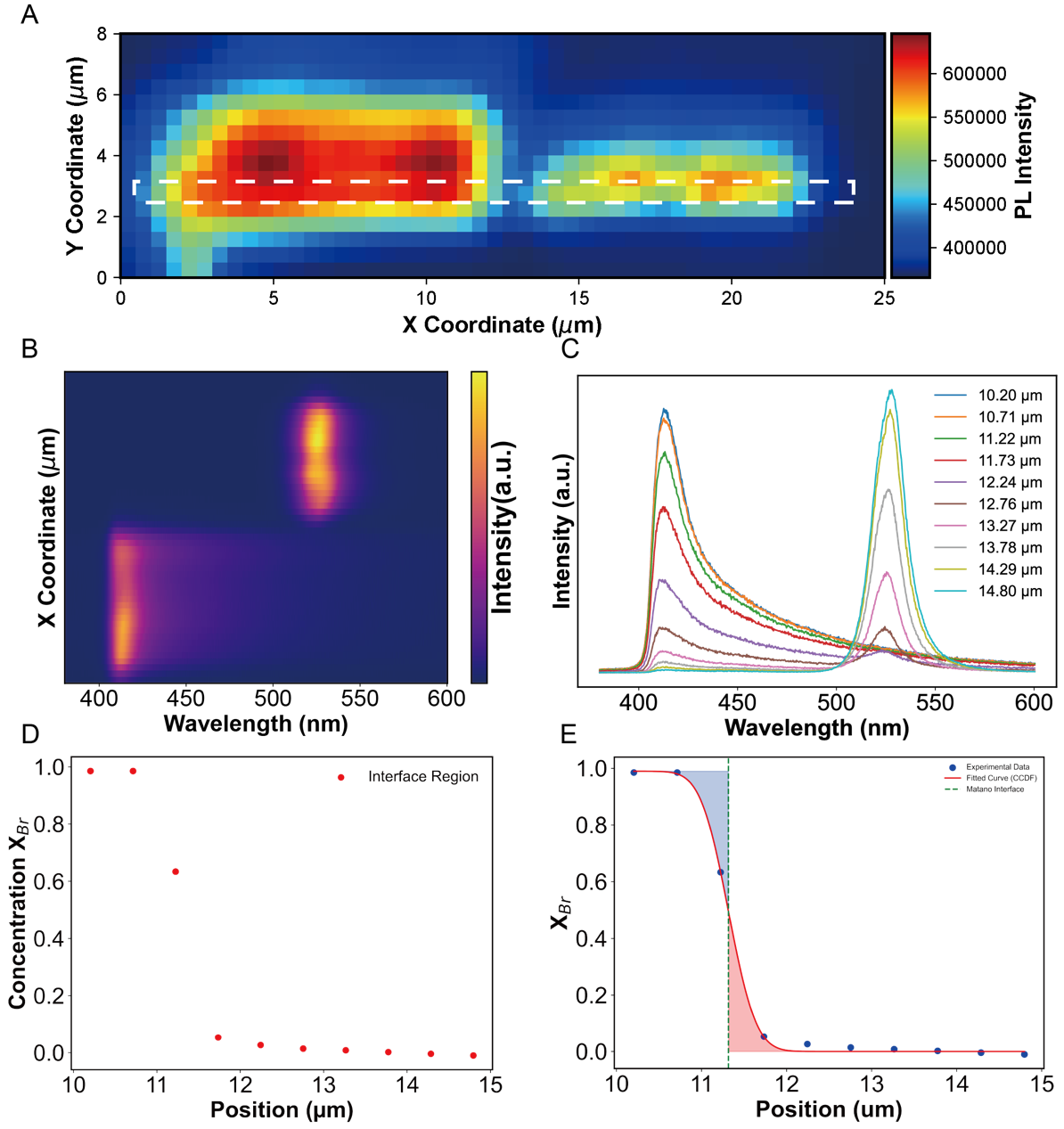


**Figure S12 | Worked example of Boltzmann–Matano (BM) analysis of halide interdiffusion in nanowire heterostructures.** **(A)** Representative PL mapping of a (PEA)₂PbI₄/(PEA)₂PbBr₄ heterostructure after thermal annealing. **(B)** Line-scan PL intensity along the junction. **(C)** Position-dependent PL spectra showing the gradual shift across the interface. **(D)** Bromide fraction profile reconstructed from PL energy via calibration. **(E)** Fitted concentration profile using a complementary cumulative distribution function (CCDF), with the Matano plane indicated. The local slope of the profile yields the concentration-dependent diffusion coefficient *D(c).*

.
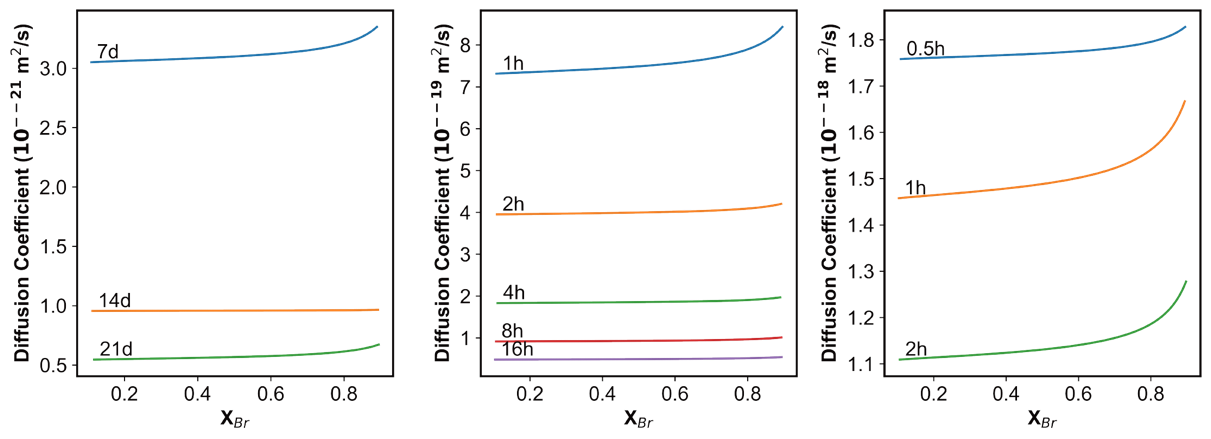


**Figure S13 | Concentration-dependent interdiffusion coefficients of (PEA)₂PbI₄/(PEA)₂PbBr₄ nanowire heterojunctions at different annealing temperatures.** Diffusion coefficients extracted using the Boltzmann–Matano (BM) method from PL-derived concentration profiles. Left: 25 °C, showing negligible diffusion even after extended aging (7–21 days). Middle: 75 °C, revealing moderate but detectable interdiffusion after 1–8 h. Right: 100 °C, where pronounced diffusion occurs within 0.5–2 h. These results highlight the strong temperature dependence of halide ion transport, with diffusion coefficients remaining more than two orders of magnitude lower than those of conventional 3D perovskites, underscoring the enhanced interfacial stability of layered nanowire heterostructures.


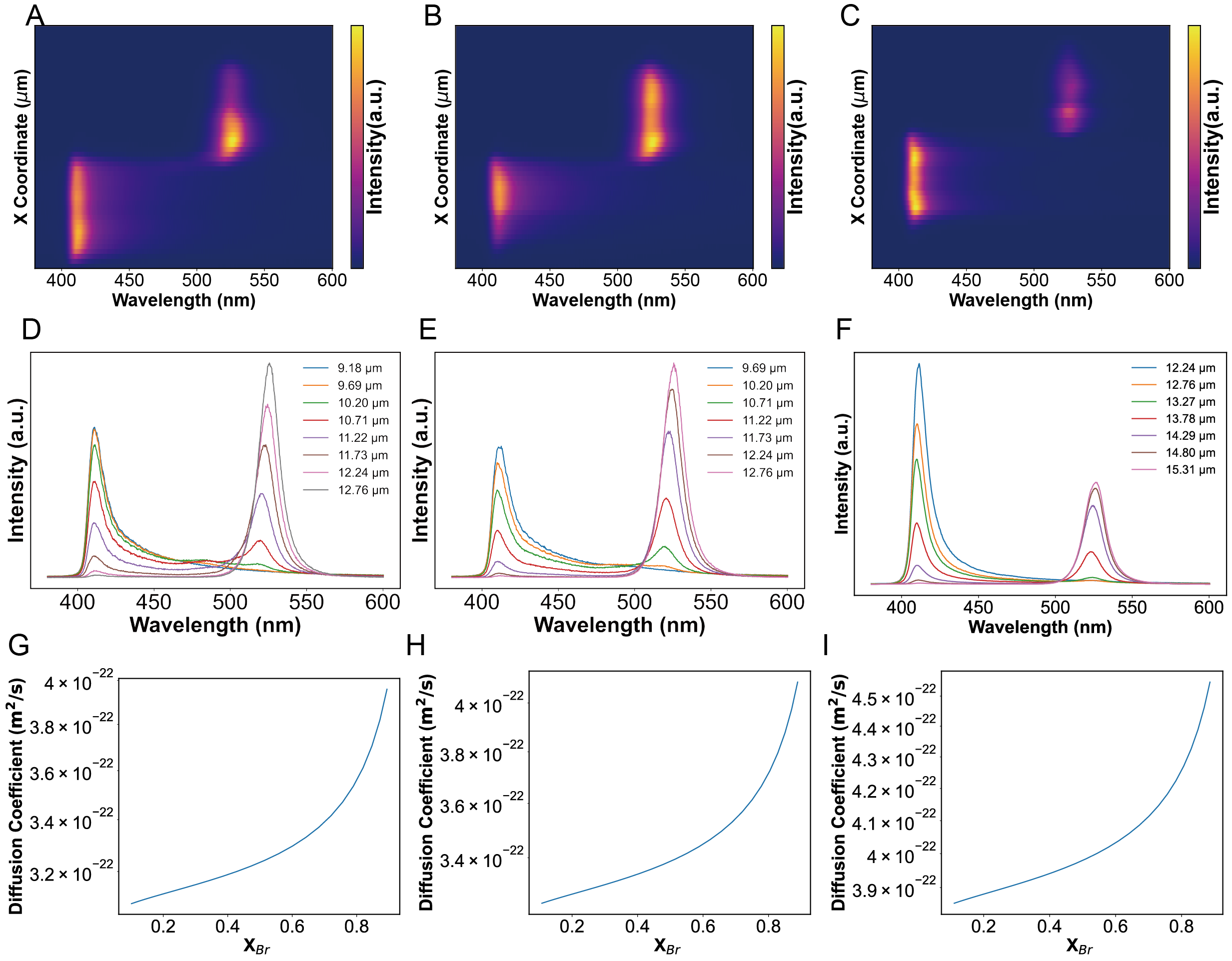


**Figure S14 | Reproducibility of diffusion analysis across independent nanowires.** PL mapping (top row), representative PL spectra along the nanowire axis (middle row), and extracted interdiffusion coefficients ~*D(c)* (bottom row) for three independently printed (PEA)₂PbI₄/(PEA)₂PbBr₄ heterostructures aged at 25 °C for 21 days. All three nanowires exhibit nearly identical concentration profiles and diffusion coefficients, with deviations within experimental uncertainty. This confirms that the reported values are robust and not artifacts of single-wire analysis


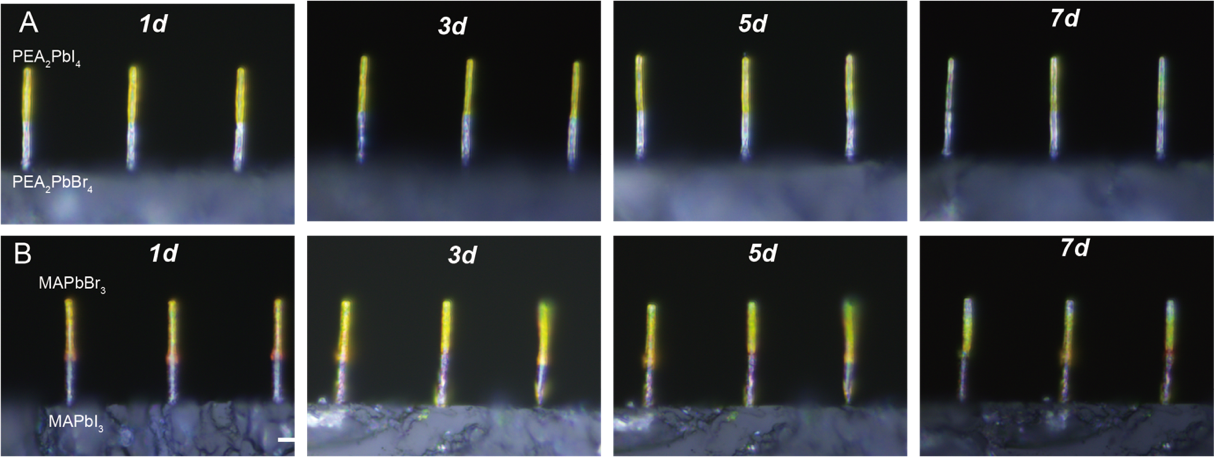


**Figure S15 | Optical images of layered vs. 3D perovskite nanowire heterojunctions under ambient storage.**

**(A) (**PEA)₂PbI₄/(PEA)₂PbBr₄ layered nanowire heterojunctions and (B) MAPbI₃/MAPbBr₃ 3D nanowire heterojunctions after one week of storage in ambient air (relative humidity 5%–10%). The layered heterostructures retain sharp emission contrast, while the 3D counterparts show pronounced degradation and color fading.

**3 Evaluation of Photodetector Performance**

**3.1 Band Energy Alignment**


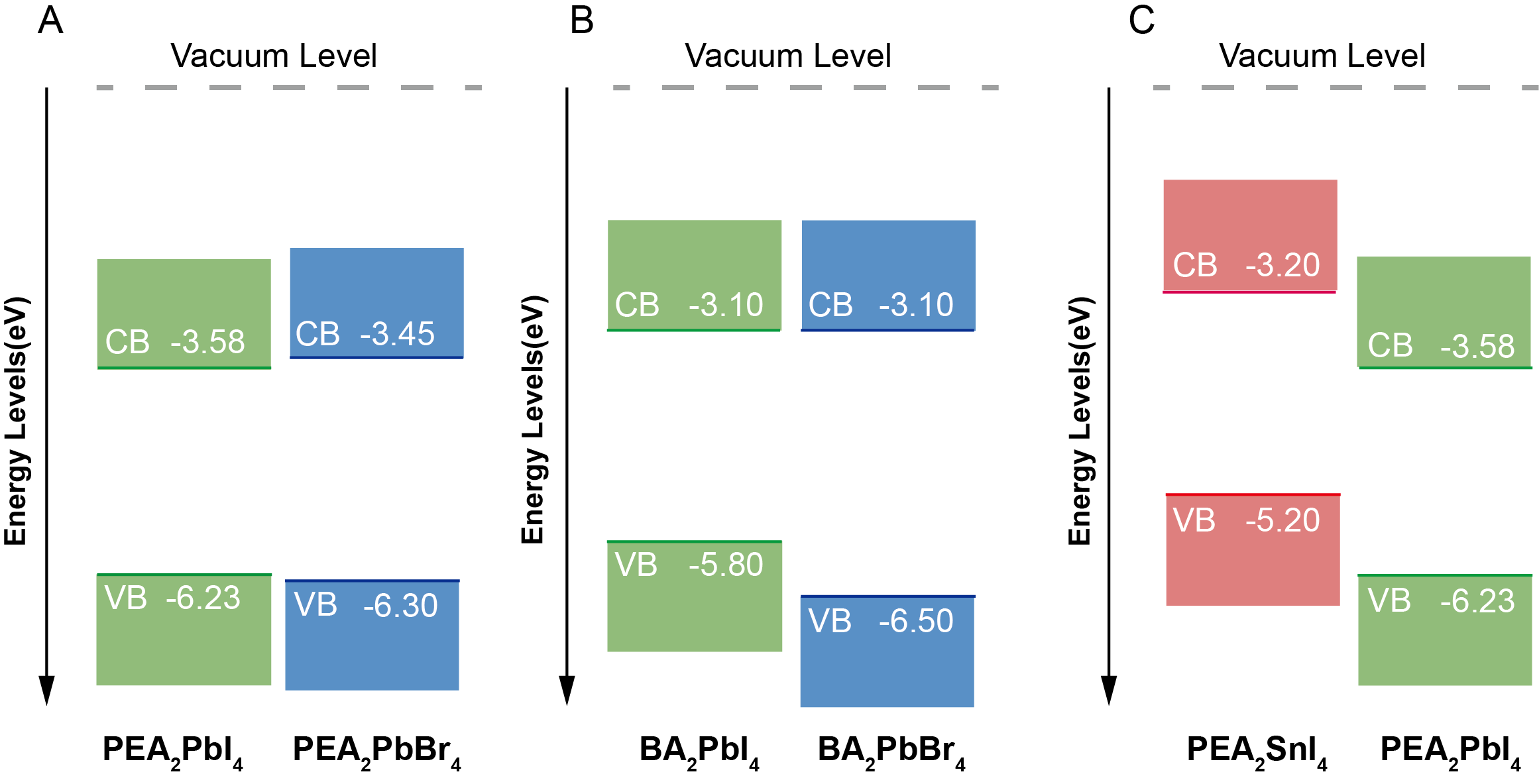


**Figure S16 | Energy level alignment of layered perovskite heterojunctions.** Schematic band alignments of representative nanowire heterostructures: **(A)** (PEA)₂PbI₄/(PEA)₂PbBr₄ showing type-I alignment, **(B)** (BA)₂PbI₄/(BA)₂PbBr₄ showing type-I alignment, and **(C)** (PEA)₂SnI₄/(PEA)₂PbI₄ showing type-II alignment with staggered bands. Values of conduction band (CB) and valence band (VB) positions were obtained from reported literature data and are provided here to clarify the expected charge separation pathways.

**
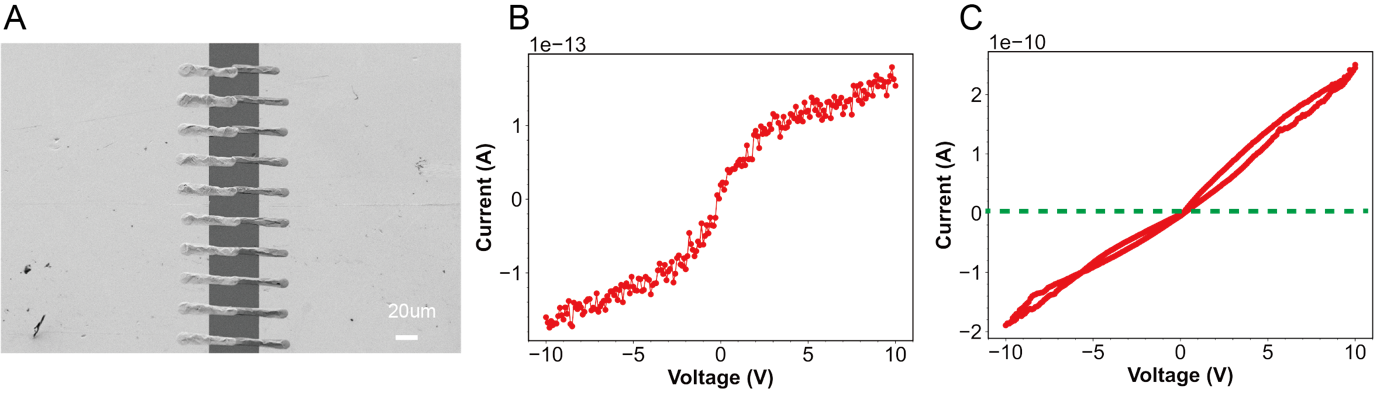
**

**Figure S17 | Device characteristics of type-I heterostructures.** (A) SEM image of a printed (PEA)₂PbBr₄/(PEA)₂PbI₄ nanowire array bridging Au electrodes (scale bar: 20 µm). (B) Dark I–V curve of the device, showing nearly linear conduction with negligible rectification. (C) Repeated I–V sweeps confirm ohmic-like behavior, in contrast to the diode-like rectification observed in type-II (PEA)₂PbI₄/(PEA)₂SnI₄ heterostructures

**3.2** **The Calculation Process**

The illuminated area is estimated from the projected area of the 10-nanowire array:

$A=NLW=10\times\left( 50\text{ }\mu\text{m} \right)\times\left( 5\text{ }\mu\text{m} \right)=2.5\times{10}^{-5}\text{ }\text{cm}^{2}\ldots$(9)

**Responsivity (R)**

Under 365 nm illumination with power density $P_{\text{opt}}=3.2\times{10}^{-3}\text{ }\text{W cm}^{-2}$, the photocurrent is

$I_{\text{ph}}=\mid I_{\text{light}}-I_{\text{dark}}\mid$...(10)

where $I_{\text{light}}\approx1.12\times{10}^{-11}\text{ A}$and $I_{\text{dark}}\approx8.10\times{10}^{-14}\text{ A}$. The responsivity is

$R=\frac{I_{\text{ph}}}{P_{\text{opt}}A}\approx1.39\times{10}^{-4}\text{ }\text{A W}^{-1}$...(11)

**Specific Detectivity (D*)**

Assuming shot-noise-limited dark-current noise, the rms noise current in bandwidth $\Delta f$is

$i_{n}=\sqrt{2qI_{\text{dark}}\Delta f}$...(12)

where $q=1.602\times{10}^{-19}\text{ C}$. The specific detectivity is

$D^{*}=\frac{R\sqrt{A}}{i_{n}}=\frac{R\sqrt{A}}{\sqrt{2qI_{\text{dark}}\Delta f}}$...(13)

Using $\Delta f=1\text{ Hz}$, we obtain $D^{*}\approx4.31\times{10}^{9}\text{ Jones}$.

**Noise-Equivalent Power (NEP)**

The NEP is defined as the incident optical power that yields SNR = 1 in a 1 Hz bandwidth:

$\text{NEP}=\frac{i_{n}}{R}$...(14)

With $\Delta f=1\text{ Hz}$, $\text{NEP}\approx1.16\times{10}^{-12}\text{ }\text{W Hz}^{-1/2}$.

**Short-Term Stability**

Short-term stability was evaluated at 0 V under 365 nm illumination (3.2 mW cm⁻²) for 28 on/off cycles (~1 s on / 1 s off) (Figure S18). For cycle $k$,

$I_{\text{ph}}\left( k \right)=I_{\text{on}}\left( k \right)-I_{\text{off}}\left( k \right)$…(15)

where $I_{\text{on}}(k)$and $I_{\text{off}}(k)$are averaged over the steady plateaus (excluding transients). The relative change is

$\Delta_{\text{rel}}=\mid\frac{I_{\text{ph}}\left( 28 \right)-I_{\text{ph}}\left( 1 \right)}{I_{\text{ph}}\left( 1 \right)}\mid\times100\%.=0.77\%<1\%$*...*(16)

**Figure S18 | Short-term operational stability of the self-powered layered nanowire heterojunction photodetector at zero bias (0 V).**

**Table S1. Summary of perovskite nanowire photodetectors**

| **Material** | **Fabrication**  **Method** | **Self**  **powered** | **Bias (V)** | **Wavelength** | **Dark Current (A)** | **Responsivity (R, A/W)** | **Detectivity (D*, Jones)** | **Ref** |
| --- | --- | --- | --- | --- | --- | --- | --- | --- |
| **(PEA)₂PbI₄/ (PEA)₂SnI₄** | **3D printing** | **Yes** | **0** | 365 nm | **8.1 × 10⁻¹⁴** | **1.39 × 10⁻⁴** | **4.3 × 10⁹** | **This Work** |
| MAPbI_3_-MAPbBr_3_ | Template-guided | Yes | 0 | 650 nm | 4.9 × 10⁻¹³ | 233 | 6.98 × 10¹³ | ^[1]^ |
| CsPbI₃ / CsPbBr₃ | Template + vapor-phase ion exchange | Yes | 0 | 650 nm | / | 1.25 × 10⁻¹ | / | ^[2]^ |
| MAPbBr₃ / MAPbBr₃₋ₓIₓ | Template-assisted assembly + anion exchange | Yes | 5.0 | 532 nm | / | 2.65 × 10² | / | ^[3]^ |
| CsPbBr_3-3x_I_3x_ | anion exchange | Yes | 2.0 | 405 nm | 10⁻¹³ | 329.36 | 1.0 × 10^11^ | ^[4]^ |
| CsPbI₃ / CsPbBr₃ | AAO template + vapor anion exchange | No | 5.0 | 405 nm | ~5.0 × 10⁻¹⁴ | 1.9 | / | ^[5]^ |
| CsPbCl_3_/CsPbI_3_ | Vapor-phase synthesis | No | 5.0 | 405 nm | ∼10⁻¹² | 49 | 1.51 × 10¹³ | ^[6]^ |
| (R-/S-α-PEA)_2_PbI_4_ | Template-assisted assembly | No | 5.0 | 505 nm | ~10⁻¹³ | 47.1 | 1.24 × 10¹³ | ^[7]^ |
| (BA)₂(MA)₃Pb₄I₁₃ (n = 4) | Template-assisted assembly | No | 5.0 | 530 nm | ∼10⁻¹² | 1.53 × 10⁴ | 7.45 × 10¹⁵ | ^[8]^ |
| (ThMA)2(MA)n-1PbnI3n+1 (n = 3) | Template-assisted assembly | No | 5.0 | 530 nm | / | 1100 | 9.1 × 10^15^ | ^[9]^ |
| (BA)₂(MA)₃Pb₄Br₁₃ (n=4) | Template-confined growth | No | 5.0 | 365 nm | 2 × 10⁻¹² | 3.5 | 1 × 10¹⁵ | ^[10]^ |

**
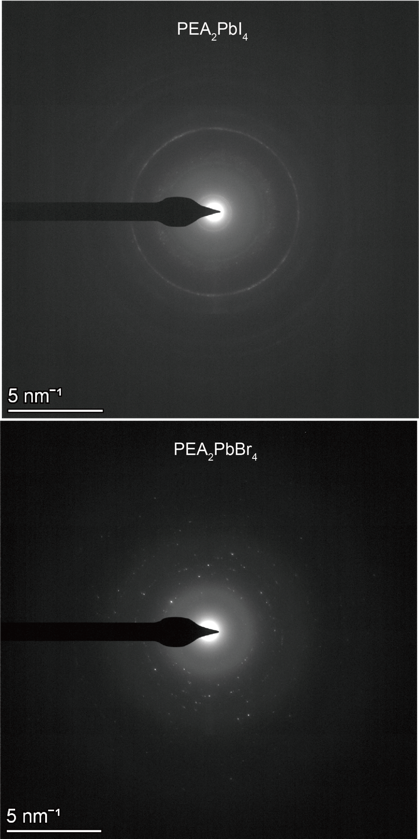
**

**Figure S19. Raw SAED patterns of layered perovskite nanowires.** Unprocessed selected-area electron diffraction (SAED) images of (top) PEA₂PbI₄ and (bottom) PEA₂PbBr₄ nanowires, shown without indexing overlays. The diffraction rings are clearly visible in both cases. The discrete spots in the PEA₂PbBr₄ pattern reflect preferred orientations within polycrystalline domains and do not indicate secondary phases.

**Reference**

1. S. X. Li, H. Xia, L. Wang, et al., “Self‐Powered and Flexible Photodetector with High Polarization Sensitivity Based on MAPbBr3–MAPbI3 Microwire Lateral Heterojunction,” Advanced Functional Materials 32 (2022): <https://doi.org/10.1002/adfm.202206999>.

2. M. Wang, W. Tian, F. Cao, et al., “Flexible and Self‐Powered Lateral Photodetector Based on Inorganic Perovskite CsPbI3–CsPbBr3 Heterojunction Nanowire Array,” Advanced Functional Materials 30 (2020): <https://doi.org/10.1002/adfm.201909771>.

3. Y. Guan, C. Zhang, Z. Liu, et al., “Single-Crystalline Perovskite p-n Junction Nanowire Arrays for Ultrasensitive Photodetection,” Advanced Materials 34 (2022): e2203201, <https://doi.org/10.1002/adma.202203201>.

4. J. Li, J. Li, M. An, et al., “Ultralong Compositional Gradient Perovskite Nanowires Fabricated by Source-Limiting Anion Exchange,” ACS Nano 18 (2024): 30978–30986, <https://doi.org/10.1021/acsnano.4c06676>.

5. Z. Zhang, N. Lamers, C. Sun, et al., “Free-Standing Metal Halide Perovskite Nanowire Arrays with Blue-Green Heterostructures,” Nano Letters 22 (2022): 2941–2947, <https://doi.org/10.1021/acs.nanolett.2c00137>.

6. Q. Lv, X. Shen, X. Li, et al., “On-Wire Design of Axial Periodic Halide Perovskite Superlattices for High-Performance Photodetection,” ACS Nano 18 (2024): 18022–18035, <https://doi.org/10.1021/acsnano.4c05205>.

7. Y. Zhao, Y. Qiu, J. Feng, et al., “Chiral 2D-Perovskite Nanowires for Stokes Photodetectors,” Journal of the American Chemical Society 143 (2021): 8437–8445, <https://doi.org/10.1021/jacs.1c02675>.

8. J. Feng, C. Gong, H. Gao, et al., “Single-crystalline layered metal-halide perovskite nanowires for ultrasensitive photodetectors,” Nature Electronics 1 (2018): 404–410, <https://doi.org/10.1038/s41928-018-0101-5>.

9. Y. Zhao, Y. Qiu, H. Gao, et al., “Layered-Perovskite Nanowires with Long-Range Orientational Order for Ultrasensitive Photodetectors,” Advanced Materials 32 (2020): e1905298, <https://doi.org/10.1002/adma.201905298>.

10. S. X. Li, G. P. Zhang, H. Xia, et al., “Template-confined growth of Ruddlesden-Popper perovskite micro-wire arrays for stable polarized photodetectors,” Nanoscale 11 (2019): 18272–18281, <https://doi.org/10.1039/c9nr05396d>.
